# Association of Average Glucose and Glycemic Variability With 28‐Day Mortality in Patients With Cardiac Arrest: A Retrospective Study

**DOI:** 10.1111/1753-0407.70179

**Published:** 2025-12-07

**Authors:** Zhenyu Shan, Yanrui Jia, Xingsheng Wang, Guyu Zhang, Chenchen Hang, Le An, Rui Shao, Ziren Tang

**Affiliations:** ^1^ Department of Emergency Medicine Beijing Chaoyang Hospital, Capital Medical University Beijing China; ^2^ Nursing Department Beijing Chaoyang Hospital, Capital Medical University Beijing China

**Keywords:** average glucose, cardiac arrest, glycaemic variability, intensive care unit, mortality

## Abstract

**Background:**

Blood glucose levels and glycemic variability are associated with poor prognosis in critically ill patients. This study aims to investigate the relationship between average glucose (AG) and glycemic variability (GV) with 28‐day mortality in patients with cardiac arrest (CA).

**Methods:**

This retrospective study extracted glucose measurements during ICU stay from all patients diagnosed with CA in the eICU Collaborative Research Database (version 2.0), the Medical Information Mart for Intensive Care III (version 1.4) and IV (version 3.1), and the Emergency Intensive Care Unit of Beijing Chaoyang Hospital. Multivariable Cox proportional hazards regression models and restricted cubic spline (RCS) analysis were used to explore the associations between AG, GV, and outcomes, with subgroup analyses performed to validate the findings.

**Results:**

In 6110 CA patients, RCS analysis revealed a nonlinear relationship between AG and 28‐day mortality (*p* for nonlinear < 0.001), whereas GV exhibited a linear association (*p* for nonlinear = 0.058). Multivariable Cox regression analysis demonstrated that AG Q4 (AG ≥ 171.75 mg/dL), GV Q4 (GV ≥ 35.8%), and Group 4 (AG ≥ 139.7 and GV ≥ 25.8%) were associated with increased 28‐day death risk (AG Q4: Hazard Ratio [95% Confidence Interval] 1.90 [1.69, 2.14], *p* < 0.001; GV Q4: 1.45 [1.30, 1.62], *p* < 0.001; Group 4: 1.80 [1.61, 2.00], *p* < 0.001), consistent across subgroup analyses.

**Conclusions:**

Our study showed that higher AG and GV were associated with mortality in CA patients. Furthermore, AG and GV were independent predictors of 28‐day mortality in these patients.

## Highlights

- This study analyzed the blood glucose measurement data during ICU hospitalization of patients diagnosed with CA from the eICU‐CRD, MIMIC‐III, MIMIC‐IV, and the Emergency Intensive Care Unit of Beijing Chaoyang Hospital.
- High average glucose and glycemic variability were independent predictors of 28‐day death risk in cardiac arrest patients.

## Graphical abstract

In this study, data from 6110 patients with cardiac arrest (CA) were analyzed, and it was found that both the average blood glucose level and blood glucose variability were associated with the prognosis of CA patients.

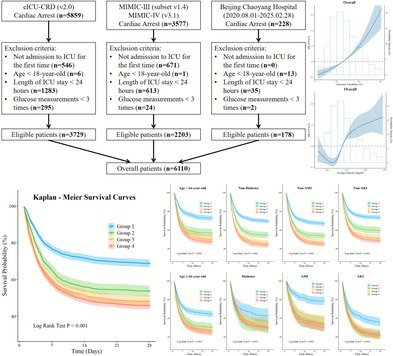

**Abbreviations**
AG: average glucose
AKI: acute kidney injury
AMI: acute myocardial infarction
BMI: body mass index
BUN: blood urea nitrogen
CA: cardiac arrest
CPR: cardiopulmonary resuscitation
CRRT: continuous renal replacement therapy
EICU: Emergency Intensive Care Unit
eICU‐CRD: eICU Collaborative Research Database
GLM: generalized linear model
GV: glycemic variability
HR: hazard ratio
ICU: Intensive Care Unit
IHCA: in‐hospital cardiac arrest
LOS: length of stay
MAP: mean arterial pressure
MIMIC: Medical Information Mart for Intensive Care
MV: mechanical ventilation
OHCA: out‐of‐hospital cardiac arrest
RCS: restricted cubic splines
ROSC: return of spontaneous circulation
TTM: target temperature management
WBC: white blood cell

## 1 Introduction

Cardiac arrest (CA) is a global health problem with a high incidence and low survival rate. In 2020, the International Liaison Committee on Resuscitation reported that the incidence of out‐of‐hospital cardiac arrest (OHCA) treated by emergency medical services globally ranges from 30.0 to 97.1 cases per 100 000 population, with only 3.1%–20.4% surviving to discharge [1]. The American Heart Association's Get With The Guidelines‐Resuscitation registry reported that over 290 000 adults experience in‐hospital cardiac arrest (IHCA) annually in the United States [2], with a 25% survival rate to discharge [3]. The priority of care after return of spontaneous circulation (ROSC) is to reduce secondary brain injury. However, the inaccuracy of neurological function prediction may lead to unnecessary withdrawal of treatment or organ donation [4], which highlights the importance of identifying and managing risk factors for poor outcomes in CA patients.

Blood glucose abnormalities, including stress‐induced hyperglycemia, hypoglycemia, and abnormal glucose variability (GV), are associated with poor clinical outcomes in various critical illnesses [5, 6]. Patients with CA and ROSC undergo anoxic‐ischemic and reperfusion phases, during which hyperglycemia plays a significant role in secondary neural injury [7]. The potential mechanisms of stress‐induced hyperglycemia include overactivation of the neuroendocrine system, interference by inflammatory cytokines, metabolic pathway disorders, and organ dysfunction [8, 9]. During glycemic control, the potential risk of hypoglycemia may increase [10], which can affect glucose energy supply and cause adverse effects. Additionally, GV, as an important indicator reflecting blood glucose fluctuations, has been shown to be associated with poor prognoses in cardiovascular and cerebrovascular diseases [11, 12, 13]. In OHCA studies, early hyperglycemia (within 36 or 48 h) and the magnitude of blood glucose changes are correlated with prognosis [5, 14], although some reports suggest that mean blood glucose levels are not an independent factor for in‐hospital mortality [15]. Therefore, the correlation between blood glucose abnormalities and prognosis requires further clarification.

Considering that previous studies have only included short‐term blood glucose indicators and there are certain differences between the GV indicators used historically and the current clinically accepted standards, this study aims to simultaneously evaluate the association of average glucose (AG) and GV with 28‐day mortality in CA patients, thereby providing better guidance for glycemic management.

## 2 Methods

### 2.1 Data Source

This study was performed with the data from the eICU Collaborative Research Database (eICU‐CRD) version 2.0, a subset of the Medical Information Mart for Intensive Care (MIMIC)‐III version 1.4, MIMIC‐IV version 3.1, and the Emergency Intensive Care Unit (EICU) of Beijing Chaoyang Hospital. Access to the dataset was obtained by completing the required training through the Collaborative Institutional Training Initiative and obtaining the relevant certification (certificate number: 61795961), as well as securing approval from the Hospital Institutional Review Board and the Ethics Committee of Beijing Chaoyang Hospital.

The eICU‐CRD is a multicenter, publicly available database of de‐identified high‐granularity healthcare data from over 200 000 ICU admissions across the United States between 2014 and 2015. MIMIC‐III and MIMIC‐IV are publicly available ICU datasets containing extensive, de‐identified data from patients admitted to the Beth Israel Deaconess Medical Center's (Boston, US) ICUs between 2001 and 2022. The databases provide extensive information, including demographic data, vital sign measurements, comprehensive laboratory test results, detailed medication records, procedural information, International Classification of Diseases (ICD) coding, and hospital length of stay (LOS).

### 2.2 Study Population

The study population consisted of patients diagnosed with CA. CA was confirmed using ICD‐9th edition code (4275) and 10th edition codes (I46, I46.2, I46.8, I46.9), and patients who were admitted to the ICU during hospitalization were included. Additionally, CA patients admitted to the EICU of Beijing Chaoyang Hospital from 2020.08.01 to 2025.02.28 were included. Exclusion criteria were as follows: (1) multiple hospital or ICU admission records; (2) patients under 18 years old; (3) patients with an ICU length of stay < 24 h; (4) patients with fewer than 3 blood glucose tests during ICU admission. Finally, 3729 patients were included from eICU‐CRD, 2203 patients from MIMIC, and 178 patients from the EICU of Beijing Chaoyang Hospital.

For the eICU‐CRD and MIMIC databases, this study was unable to determine the glucose intervention protocols for this subset of patients. For patients from the EICU of Beijing Chaoyang Hospital, all post‐CA patients were managed in accordance with the American Heart Association guidelines [16], aligned with the treatment recommendations of the International Liaison Committee on Resuscitation [17] and practical clinical considerations, and updated following revisions to the guidelines and treatment recommendations. Blood glucose monitoring was primarily performed via point‐of‐care testing at the bedside, with a routine frequency of every 4 h, which could be adjusted based on actual clinical considerations. Blood glucose was controlled through glucose injection and continuous insulin infusion, with a target glucose range of 8–10 mmol/L (144–180 mg/dL) for all patients.

### 2.3 Data Extraction and Definitions

In this study, Structured Query Language was used with Navicat Premium software (version 16) to extract demographic characteristics, vital signs, comorbidities, laboratory tests, and treatment data of the study subjects. For vital signs and laboratory tests, the first data after ICU admission were extracted. Vasoactive‐inotropic drugs refer to the use of vasoactive drugs or positive inotropic drugs, including epinephrine, norepinephrine, phenylephrine, dopamine, dobutamine, vasopressin, milrinone, or digoxin. Variables with missing values exceeding 20% were excluded, and variables with missing values less than 20% were imputed using multiple imputation methods.

Obtain all blood glucose levels during the patient's ICU stay, including bedside and laboratory test results. All values should be measured before the occurrence of the endpoint event, and at least three measurements should be performed. The same blood glucose value at the same time is considered one test. AG is defined as the mean of all blood glucose measurements during ICU admission, and GV is evaluated using the coefficient of variation of blood glucose, defined as the ratio of the standard deviation to the mean of all blood glucose measurements during ICU admission (standard deviation [SD]/mean × 100%). Calculate the interquartile ranges (IQR) of AG and GV through the Lower Quartile (Q1) and Upper Quartile (Q3). In this study, values below Q1–1.5 × IQR or above Q3 + 1.5 × IQR were regarded as outliers. These outliers were subjected to re‐evaluation before being included in the statistical analysis and were excluded when plotting the restricted cubic splines curve.

### 2.4 Study Endpoint

The primary endpoint of this study was 28‐day all‐cause mortality after ICU admission. Secondary endpoints included the LOS in the ICU.

### 2.5 Statistical Analysis

Categorical variables were described as frequencies with percentages (%). Continuous variables conforming to the normal distribution were described as means with SD; otherwise, they were described as medians with IQR.

**FIGURE 1:**
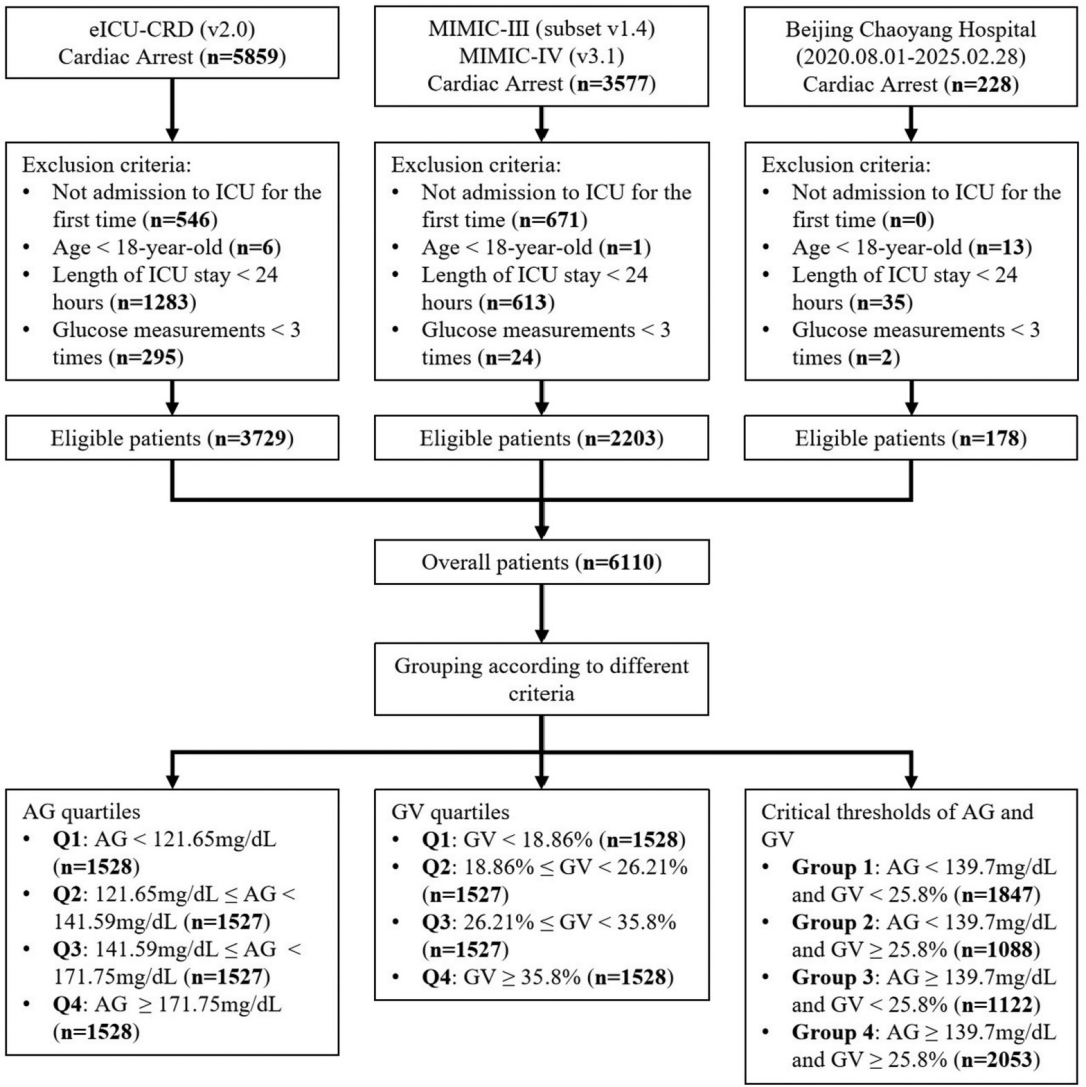
Flowchart of study.

The comparison of categorical variables used the Chi‐square test or Fisher's exact test. The comparison of continuous variables used the Student's *t*‐test or the Mann–Whitney *U* test (two groups), and the Analysis of Variance or the Kruskal–Wallis test (more than two groups). RCS were used to evaluate the relationship between AG, GV, and 28‐day survival, and critical thresholds were determined (AG: 139.7 mg/dL, GV: 25.8%). Based on the quartiles of AG and GV, as well as the critical thresholds from the RCS analysis of AG and GV, multiple groupings were performed, including: AG quartiles: Q1 (< 121.65 mg/dL), Q2 (121.65 mg/dL to < 141.59 mg/dL), Q3 (141.59 mg/dL to < 171.75 mg/dL), Q4 (≥ 171.75 mg/dL); GV quartiles: Q1 (< 18.86%), Q2 (18.86% to < 26.21%), Q3 (26.21% to < 35.8%), Q4 (≥ 35.8%). AG and GV: Group 1 (AG < 139.7 mg/dL and GV < 25.8%), Group 2 (AG < 139.7 mg/dL and GV ≥ 25.8%), Group 3 (AG ≥ 139.7 mg/dL and GV < 25.8%), Group 4 (AG ≥ 139.7 mg/dL and GV ≥ 25.8%) (Figure 1).

Cox proportional hazards model analysis was used to analyze the association between different groupings and 28‐day mortality, and variables were included in a multivariate model for adjustment. The adjusted variables in different models are as follows: Model 1: unadjusted. Model 2: Model 1 + Gender, Age, Race, and Body mass index (BMI). Model 3: Model 2 + Mean arterial pressure (MAP), Temperature, Heart rate, and Respiratory rate. Model 4: Model 3 + Hypertension, Diabetes, Cerebral infarction, Acute myocardial infarction (AMI), Acute kidney injury (AKI), Liver cirrhosis, Chronic kidney disease, and Malignant tumor. Model 5: Model 4 + White blood cell (WBC) counts, Hemoglobin, Platelet counts, Blood urea nitrogen (BUN), Creatinine, Total calcium, Sodium, Potassium, Chloride, and Anion gap. Model 6: Model 5 + Mechanical ventilation (MV), Continuous renal replacement therapy (CRRT), Vasoactive‐inotropic drugs, and Insulin. Considering the multiple testing, we used the Holm‐Bonferroni method to adjust the raw *p*‐values. The Kaplan–Meier method was used to construct survival curves for patients in different groups, and the log‐rank test was applied to assess differences between groups. In addition, this study performed multivariate RCS analysis on AG and GV to identify their potential nonlinear relationships with 28‐day survival. The optimal number of knots for the RCS model was determined by the minimum Akaike information criterion. Due to the non‐normal distribution of the data, natural logarithmic transformation was applied to AG and GV before they were used as continuous variables in multivariate Cox regression analysis. In terms of sensitivity analysis, we conducted analyses across different time periods after ICU admission and analyzed the three databases separately to verify the robustness of the initial conclusions. When conducting subgroup analysis, we grouped the data based on categorical variables or the thresholds of continuous variables derived from RCS analysis. Forest plots were drawn to display the results of subgroup analyses and identify potential interaction effects. For subgroups with significant interaction effects, Kaplan–Meier survival curves were separately plotted, and log‐rank tests were conducted.

The Pearson or Spearman correlation test was used to analyze the correlation between AG, GV (as continuous variables), and the LOS in the ICU. A generalized linear model (GLM) was then employed to determine their relationship after adjusting for other variables. Additionally, subgroup analyses were performed to explore the associations of AG and GV with LOS in 28‐day survivors and nonsurvivors separately.

All the statistical analyses were performed using the R software version 4.3.1 (R Development Core Team, 2023) with RStudio version 2023.12.1. All tests were two‐tailed, and a *p*‐value of less than 0.05 was considered statistically significant.

## 3 Results

### 3.1 Baseline Characteristics

This study enrolled a total of 6110 CA patients, including 3729 cases from the eICU‐CRD database, 2203 cases from the MIMIC database, and 178 cases from Beijing Chaoyang Hospital (Figure 1). Among overall patients, 2421 (39.62%) were female, with a median age of 66 (55–76) years (Table 1). A total of 2782 (45.53%) patients died within 28 days, and the median length of ICU stay was 102.3 (58.0–194.7) hours. Multiple groupings were performed based on the quartiles of AG and GV, as well as their critical thresholds (Figure 1). Comparisons between different groups are shown in Tables 1, S1 and S2.

**TABLE 1:** Baseline characteristics of patients with cardiac arrest according to average glucose and glycemic variability.

Variable	Overall (*n* = 6110)	Group 1 (*n* = 1847)	Group 2 (*n* = 1088)	Group 3 (*n* = 1122)	Group 4 (*n* = 2053)	*p*
Demographics						
Female, *n* (%)	2421 (39.62%)	661 (35.79%)	447 (41.08%)	420 (37.43%)	893 (43.5%)	< 0.001
Age, year	66 (55, 76)	63 (52, 75)	64 (53, 76)	68.5 (59, 78)	67 (56, 75)	< 0.001
Race, *n* (%)						< 0.001
White	3997 (65.42%)	1257 (68.06%)	694 (63.79%)	772 (68.81%)	1274 (62.06%)	
Black	788 (12.9%)	219 (11.86%)	172 (15.81%)	106 (9.45%)	291 (14.17%)	
Other/Unknown	1325 (21.69%)	371 (20.09%)	222 (20.4%)	244 (21.75%)	488 (23.77%)	
BMI, kg/m^2^	28.04 (24.24, 33.54)	27.36 (23.79, 32.65)	26.32 (22.89, 31.44)	29.27 (25.72, 34.89)	28.73 (24.75, 34.8)	< 0.001
Vital signs						
MAP, mmHg	82 (69, 98)	83.17 (71, 98.16)	82 (68, 97)	82 (69, 97.25)	82 (68.5, 97)	0.017
Temperature, °C	36.5 (35.56, 36.94)	36.56 (35.78, 37)	36.39 (35.2, 36.89)	36.6 (35.84, 37)	36.4 (35.3, 36.89)	< 0.001
Heart rate, bpm	89 (75, 106)	88 (73, 104)	89 (76, 106)	88 (75, 105)	91 (76, 109)	< 0.001
Respiratory rate, bpm	20 (16, 24)	19 (16, 23)	20 (16, 24)	20 (16, 24)	20 (16, 25)	< 0.001
Comorbidities						
Hypertension, *n* (%)	5238 (85.73%)	1552 (83.98%)	921 (84.73%)	998 (88.79%)	1767 (86.15%)	0.002
Diabetes, *n* (%)	1454 (23.8%)	166 (8.98%)	211 (19.41%)	304 (27.05%)	773 (37.69%)	< 0.001
Cerebral infarction, *n* (%)	219 (3.58%)	40 (2.16%)	36 (3.31%)	51 (4.54%)	92 (4.49%)	< 0.001
Acute myocardial infarction, *n* (%)	1071 (17.53%)	316 (17.1%)	166 (15.27%)	189 (16.81%)	400 (19.5%)	0.018
Acute kidney injury, *n* (%)	2051 (33.57%)	468 (25.32%)	376 (34.59%)	380 (33.81%)	827 (40.32%)	< 0.001
Liver cirrhosis, *n* (%)	157 (2.57%)	49 (2.65%)	37 (3.4%)	34 (3.02%)	37 (1.8%)	0.04
Chronic kidney disease, *n* (%)	1178 (19.28%)	290 (15.69%)	264 (24.29%)	195 (17.35%)	429 (20.92%)	< 0.001
Malignant tumor, *n* (%)	419 (6.86%)	131 (7.09%)	64 (5.89%)	95 (8.45%)	129 (6.29%)	0.059
Laboratory measurements						
WBC counts, K/μL	13.4 (9.6, 19.12)	12.2 (9.1, 17.1)	12.9 (8.95, 19)	13.4 (9.7, 18.9)	14.8 (10.3, 21.19)	< 0.001
Hemoglobin, g/dL	11 (9.3, 13.3)	11.3 (9.5, 13.33)	10.65 (8.9, 13.1)	11.15 (9.4, 13.4)	11 (9.3, 13.3)	< 0.001
Platelet counts, K/μL	195 (145, 260)	191 (147, 255)	186 (132, 252)	198 (148, 257)	203 (149.75, 269)	< 0.001
BUN, mg/dL	23 (16, 39)	19 (14, 32)	23 (16, 41)	25 (18, 42)	27 (19, 44)	< 0.001
Creatinine, mg/dL	1.2 (0.9, 2.12)	1 (0.8, 1.7)	1.32 (0.95, 2.52)	1.24 (0.9, 2.06)	1.4 (1.04, 2.34)	< 0.001
Total calcium, mg/dL	8.2 (7.6, 8.7)	8.2 (7.7, 8.7)	8.1 (7.4, 8.7)	8.2 (7.6, 8.7)	8.1 (7.6, 8.7)	0.005
Sodium, mmol/L	139 (136, 142)	139 (136, 142)	139 (136, 142)	139 (136, 142)	138 (135, 142)	< 0.001
Potassium, mmol/L	4.1 (3.7, 4.7)	4 (3.7, 4.5)	4.1 (3.6, 4.7)	4.1 (3.7, 4.7)	4.1 (3.6, 4.8)	< 0.001
Chloride, mmol/L	104 (100, 108.13)	105 (101, 109)	105 (100, 109)	104 (100, 108)	103 (99, 108)	< 0.001
Anion gap, mmol/L	14 (11, 18)	12.1 (10, 16)	15 (11, 19)	14 (11, 17.2)	15 (12, 20)	< 0.001
Average glucose, mg/dL	139.93 (121.65, 171.74)	119.52 (108.59, 129.4)	123.44 (112.52, 132.1)	162.18 (150.3, 188.15)	171.12 (154.57, 201.01)	< 0.001
SD of glucose, mg/dL	36.13 (24.8, 57.49)	21.22 (15.88, 26.11)	39.72 (34.89, 49.34)	32.37 (25.98, 39.49)	64.54 (53.03, 84.9)	< 0.001
Glycemic variability, %	25.84 (18.89, 35.8)	17.97 (13.87, 21.82)	32.55 (28.96, 39.93)	19.59 (15.98, 22.68)	36.37 (30.99, 45.42)	< 0.001
Treatments						
Mechanical ventilation, *n* (%)	4491 (73.5%)	1264 (68.44%)	802 (73.71%)	837 (74.6%)	588 (77.35%)	< 0.001
CRRT, *n* (%)	686 (11.23%)	140 (7.58%)	151 (13.88%)	113 (10.07%)	282 (13.74%)	< 0.001
Vasoactive‐inotropic drugs, *n* (%)	4315 (70.62%)	1165 (63.08%)	832 (76.47%)	763 (68%)	1555 (75.74%)	< 0.001
Insulin, *n* (%)	4016 (65.73%)	983 (53.22%)	708 (65.07%)	759 (67.65%)	1566 (76.28%)	< 0.001
Outcomes						
28‐day mortality, *n* (%)	2782 (45.53%)	579 (31.35%)	505 (46.42%)	584 (52.05%)	1114 (54.26%)	< 0.001
Length of ICU stay, hours	102.26 (57.96, 194.67)	100.65 (60.16, 191.94)	100.02 (65.26, 194.12)	89.21 (47.97, 186.32)	106.99 (59.59, 207.09)	< 0.001

*Note:* Data are shown as median (IQR) or *n* (%).

Group 1: AG < 139.7 mg/dL and GV < 25.8%; Group 2: AG < 139.7 mg/dL and GV ≥ 25.8%; Group 3: AG ≥ 139.7 mg/dL and GV < 25.8%; Group 4: AG ≥ 139.7 mg/dL and GV ≥ 25.8%.

*p*‐value indicates the chi‐square test or Kruskal–Wallis test.

Abbreviations: AG: average glucose, BMI: body mass index, BUN: blood urea nitrogen, CRRT: continuous renal replacement therapy, GV: glycemic variability, ICU: Intensive Care Unit, MAP: mean arterial pressure, WBC: white blood cell.

Patients in the higher AG and GV groups had a higher proportion of females and older age, as well as a higher prevalence of comorbidities, including diabetes, cerebral infarction, AMI, and AKI (all *p* < 0.05). Additionally, these patients had higher counts of WBC and platelets, as well as elevated levels of BUN and creatinine (all *p* < 0.05). They were more likely to receive MV, CRRT, vasoactive‐inotropic drugs, and insulin therapy (all *p* < 0.05). Ultimately, these patients exhibited a higher 28‐day mortality rate (*p* < 0.05, Table 1, Tables S1 and S2).

### 3.2 Association Between AG, GV, and 28‐Day Mortality

**FIGURE 2:**
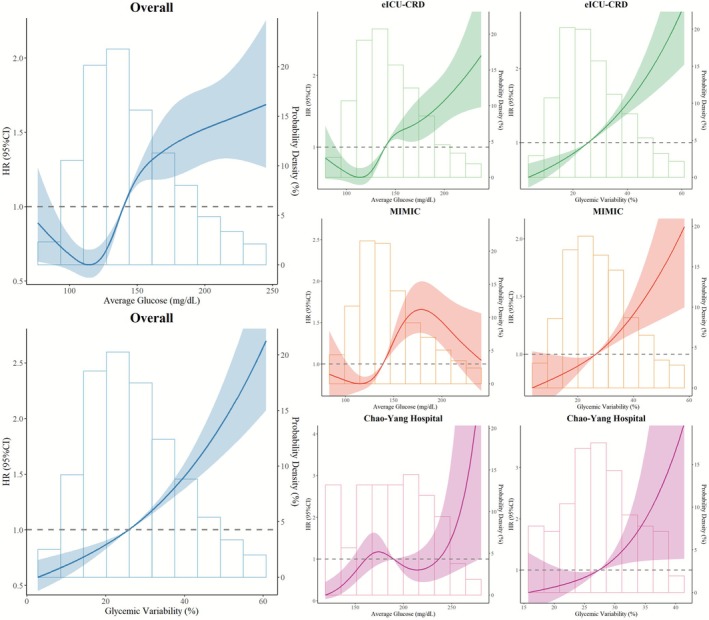
Restricted cubic spline analysis of the relationship between average glucose, glycemic variability, and 28‐day mortality in different databases. eICU‐CRD: eICU Collaborative Research Database, MIMIC: Medical Information Mart for Intensive Care‐III and IV.

**FIGURE 3:**
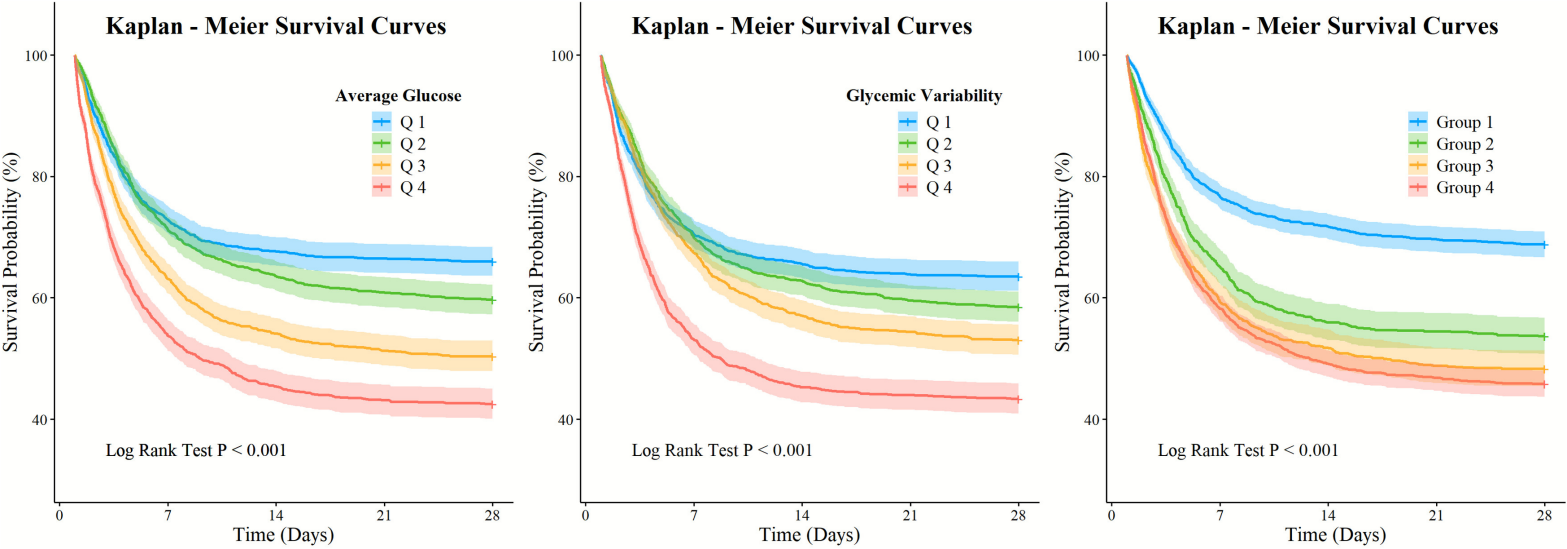
Kaplan–Meier curves for 28‐day mortality were plotted according to average glucose and glycemic variability grouping.

The number of 28‐day deaths and mortality rates in different AG groups is as follows: Q1, 522 (34.16%); Q2, 619 (40.54%); Q3, 762 (49.9%); Q4, 879 (57.53%) (*p* < 0.001, Table S1). Kaplan–Meier analysis (Figure 2) showed that the 28‐day mortality rate significantly increased with the elevation of AG across the four groups, in the order of Q1 < Q2 < Q3 < Q4. Multivariate Cox proportional hazards model analysis (Table 2 and Table S3) indicated that higher AG levels (Q3 and Q4) were significantly associated with a 28‐day mortality risk (Q3: HR [95% CI]: 1.46 [1.30, 1.64]; Q4: 1.90 [1.69, 2.14]; all *p* < 0.001). Additionally, ln(AG) was independently associated with an increased 28‐day death risk (HR [95% CI]: 1.53 [1.42, 1.66]; *p* < 0.001). RCS analysis (Figure 3) showed a nonlinear association between AG and 28‐day mortality risk in the overall patient population (*p*‐overall < 0.001; *p*‐nonlinear < 0.001). Specifically, patients had a lower death risk when AG was less than 139.7 mg/dL, and this threshold was similar across different patient groups and for ln(AG) (eICU‐CRD: 139.3 mg/dL, MIMIC: 138.8 mg/dL, Chaoyang Hospital: 145.9 mg/dL, ln(AG): 4.95 [141.2 mg/dL], Figures 3, S1 and S2). This nonlinear relationship remained significant after adjusting for multiple factors (Figures S1 and S2).

**TABLE 2:** Cox proportional hazards models analysis for different groups and 28‐day mortality in cardiac arrest patients.

	Model 1			Model 2			Model 3		
	HR (95% CI)	*p*	*p*‐adjusted	HR (95% CI)	*p*	*p*‐adjusted	HR (95% CI)	*p*	*p*‐adjusted
Average glucose									
Q1	1	—	—	1	—	—	1	—	—
Q2	1.19 (1.05, 1.34)	0.004	0.004	1.16 (1.03, 1.30)	0.013	0.051	1.13 (1.00, 1.27)	0.044	0.133
Q3	1.60 (1.43, 1.78)	< 0.001	< 0.001	1.56 (1.39, 1.74)	< 0.001	< 0.001	1.52 (1.36, 1.70)	< 0.001	< 0.001
Q4	2.07 (1.86, 2.31)	< 0.001	< 0.001	2.01 (1.81, 2.25)	< 0.001	< 0.001	1.96 (1.76, 2.19)	< 0.001	< 0.001
Glycemic variability									
Q1	1	—	—	1	—	—	1	—	—
Q2	1.13 (1.01, 1.27)	0.032	0.032	1.12 (1.00, 1.26)	0.050	0.151	1.10 (0.98, 1.23)	0.101	0.318
Q3	1.32 (1.18, 1.47)	< 0.001	< 0.001	1.29 (1.16, 1.44)	< 0.001	< 0.001	1.25 (1.12, 1.40)	< 0.001	< 0.001
Q4	1.85 (1.66, 2.06)	< 0.001	< 0.001	1.83 (1.65, 2.04)	< 0.001	< 0.001	1.75 (1.57, 1.95)	< 0.001	< 0.001
Average glucose and glycemic variability									
Group 1	1	—	—	1	—	—	1	—	
Group 2	1.66 (1.47, 1.87)	< 0.001	< 0.001	1.64 (1.46, 1.85)	< 0.001	< 0.001	1.59 (1.41, 1.80)	< 0.001	< 0.001
Group 3	1.99 (1.77, 2.23)	< 0.001	< 0.001	1.95 (1.74, 2.19)	< 0.001	< 0.001	1.94 (1.73, 2.18)	< 0.001	< 0.001
Group 4	2.09 (1.89, 2.31)	< 0.001	< 0.001	2.05 (1.85, 2.27)	< 0.001	< 0.001	1.99 (1.80, 2.20)	< 0.001	< 0.001

	Model 4			Model 5			Model 6		
	HR (95% CI)	*p*	*p*‐adjusted	HR (95% CI)	*p*	*p*‐adjusted	HR (95% CI)	*p*	*p*‐adjusted
Average glucose									
Q1	1	—	—	1	—	—	1	—	—
Q2	1.14 (1.01, 1.28)	0.030	0.162	1.12 (0.99, 1.26)	0.068	0.747	1.08 (0.96, 1.22)	0.183	> 0.999
Q3	1.57 (1.40, 1.76)	< 0.001	< 0.001	1.50 (1.34, 1.68)	< 0.001	< 0.001	1.46 (1.30, 1.64)	< 0.001	< 0.001
Q4	2.06 (1.83, 2.31)	< 0.001	< 0.001	1.94 (1.72, 2.18)	< 0.001	< 0.001	1.90 (1.69, 2.14)	< 0.001	< 0.001
Glycemic variability									
Q1	1	—	—	1	—	—	1	—	—
Q2	1.09 (0.97, 1.22)	0.160	0.704	1.06 (0.94, 1.19)	0.329	> 0.999	1.02 (0.91, 1.15)	0.726	> 0.999
Q3	1.23 (1.09, 1.37)	< 0.001	0.006	1.14 (1.02, 1.28)	0.020	0.322	1.09 (0.97, 1.22)	0.152	> 0.999
Q4	1.73 (1.55, 1.93)	< 0.001	< 0.001	1.56 (1.39, 1.74)	< 0.001	< 0.001	1.45 (1.30, 1.63)	< 0.001	< 0.001
Group 1	1	—	—	1	—	—	1	—	—
Group 2	1.58 (1.39, 1.77)	< 0.001	< 0.001	1.45 (1.29, 1.64)	< 0.001	< 0.001	1.39 (1.23, 1.57)	< 0.001	< 0.001
Group 3	1.95 (1.74, 2.20)	< 0.001	< 0.001	1.88 (1.67, 2.12)	< 0.001	< 0.001	1.88 (1.67, 2.11)	< 0.001	< 0.001
Group 4	2.04 (1.83, 2.26)	< 0.001	< 0.001	1.86 (1.67, 2.07)	< 0.001	< 0.001	1.80 (1.61, 2.00)	< 0.001	< 0.001

*Note:* Average glucose Q1: AG < 121.65 mg/dL, Q2: 121.65 mg/dL≤ AG < 141.59 mg/dL, Q3: 141.59 mg/dL≤ AG < 171.75 mg/dL, Q4: AG ≥ 171.75 mg/dL.

Glycemic variability Q1: GV < 18.86%, Q2: 18.86% ≤ GV < 26.21%, Q3: 26.21% ≤ GV < 35.8%, Q4: GV ≥ 35.8%.

Group 1: AG < 139.7 mg/dL and GV < 25.8%; Group 2: AG < 139.7 mg/dL and GV ≥ 25.8%; Group 3: AG ≥ 139.7 mg/dL and GV < 25.8%; Group 4: AG ≥ 139.7 mg/dL and GV ≥ 25.8%.

Model 1: unadjusted.

Model 2: Model 1 + Gender, Age, Race, and BMI.

Model 3: Model 2 + MAP, Temperature, Heart rate, and Respiratory rate.

Model 4: Model 3 + hypertension, diabetes, cerebral infarction, acute myocardial infarction, acute kidney injury, liver cirrhosis, chronic kidney disease, and malignant tumor.

Model 5: Model 4 + WBC counts, hemoglobin, platelet counts, BUN, creatinine, total calcium, sodium, potassium, chloride, and anion gap.

Model 6: Model 5 + mechanical ventilation, CRRT, vasoactive‐inotropic drugs, and insulin.

Abbreviations: AG: average glucose, BMI: body mass index, BUN: blood urea nitrogen, CRRT: continuous renal replacement therapy, GV: glycemic variability, HR: hazard ratio, MAP: mean arterial pressure, WBC: white blood cell.

The 28‐day mortality counts and rates across different GV groups are as follows: Q1 with 560 deaths (36.65%); Q2 with 637 deaths (41.72%); Q3 with 718 deaths (47.02%); Q4 with 867 deaths (56.74%) (*p* < 0.001, Table S2). Kaplan–Meier analysis (Figure 2) demonstrated that the 28‐day mortality rates significantly increased with rising GV levels across the four groups, in the order of Q1 < Q2 < Q3 < Q4. Multivariate Cox proportional hazards model analysis (Table 2 and Table S3) showed that GV in the Q4 group was significantly associated with a 28‐day mortality risk (HR [95% CI]: 1.45 [1.30, 1.62]; *p* < 0.001). Ln(GV) was independently associated with an increased 28‐day mortality risk (HR [95% CI]: 1.33 [1.23, 1.45]; *p* < 0.001). RCS analysis (Figures 3, S1 and S2) revealed a linear association between GV and 28‐day mortality risk in the overall patient population (*p*‐overall < 0.001; *p*‐nonlinear = 0.058). Specifically, patients had a lower mortality risk when GV was below 25.8%, and this threshold was consistent across different patient cohorts and for ln(GV) (eICU‐CRD: 25.5%, MIMIC: 26.4%, Chaoyang Hospital: 27.4%, ln(GV): 3.27 [26.3%], Figures 3, S1 and S2).

The 28‐day mortality rates across different AG combined with GV groups are as follows: Group 1, 31.35%; Group 2, 46.42%; Group 3, 52.05%; Group 4, 54.26% (*p* < 0.001, Table 1). Kaplan–Meier analysis (Figure 2) showed that the 28‐day mortality rates increased with the elevation of AG and GV across the four groups, in the order of Group 1 < Group 2 < Group 3 < Group 4. Results from the multivariate Cox proportional hazards model analysis (Table 2) indicated that the 28‐day mortality risks in all other groups were significantly higher than those in Group 1 (Group 2: HR [95% CI]: 1.39 [1.23, 1.57]; Group 3: 1.88 [1.67, 2.11]; Group 4: 1.80 [1.61, 2.00]; all *p* < 0.001).

### 3.3 Sensitivity Analysis

In this study, sensitivity analyses were conducted for different ICU stay durations and different datasets. The results showed that AG and GV on the first day, first 2 days, and first 3 days after ICU admission were all associated with patient prognosis, with patients having higher AG and GV showing poorer prognosis (Figure S3). Among them, AG exhibited a nonlinear relationship (all *p*‐overall < 0.001; all *p*‐nonlinear < 0.001), while GV showed a linear relationship (all *p*‐overall < 0.001; all *p*‐nonlinear > 0.05).

In the three databases, Kaplan–Meier analyses consistently showed that the proportion of patients who died within 28 days was significantly higher in the groups with higher AG and GV (all *p* < 0.05) (Figure S4). After adjusting for multiple variables, the eICU‐CRD and MIMIC databases still indicated that Groups 3 and 4 were associated with an increased risk of mortality (Table S4).

In the Beijing Chaoyang Hospital Database, we further adjusted for CA‐specific variables, including the time from CA to ROSC, etiologies, bystander cardiopulmonary resuscitation (CPR), and initial shockable rhythm. Additionally, neurological prognosis was incorporated as an outcome measure, which was assessed using the Cerebral Performance Category (CPC) scale (CPC 1–2 indicating a favorable prognosis and CPC 3–5 indicating a poor prognosis) [18]. The results demonstrated that the groups with higher AG and higher GV had a higher proportion of patients with 28‐day mortality and poor neurological prognosis at discharge (all *p* < 0.05) (Figure S4, Tables S5–S7). Moreover, after adjusting for multiple variables, Group 4 was associated with a high risk of mortality (Table S6).

### 3.4 Subgroup Analysis

**FIGURE 4:**
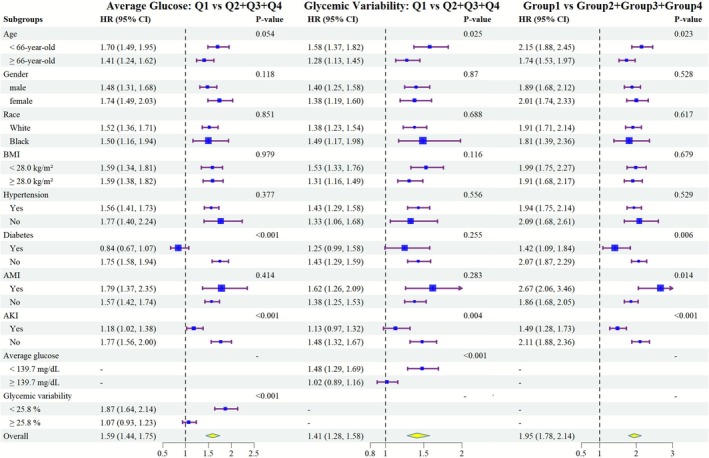
Subgroup analysis of 28‐day death risk according to average glucose and glycemic variability grouping.

Figures 4, S5–S10 display subgroup analyses based on demographic data and comorbidities. Regarding the association between different AG groups and 28‐day mortality, results showed that higher AG groups were associated with increased mortality risk across all subgroups, except for patients with diabetes (HR [95% CI] 0.84 [0.67, 1.07], *p* = 0.159) and those with GV ≥ 28.5% (HR [95% CI] 1.07 [0.93, 1.23], *p* = 0.337). No significant interactions were observed between AG and age, gender, race, BMI, or hypertension (all *p* > 0.05). Notably, in subgroups of patients without diabetes, without AKI, or with GV < 25.8%, higher AG groups exhibited more pronounced mortality risks (Figures 4, S6 and S7). Kaplan–Meier analysis across subgroups revealed that the AG Q4 group consistently had the highest 28‐day mortality risk (Figure S8). Notably, in diabetic patients, the 28‐day death risk in the AG Q1 group was higher (HR [95% CI] 1.35 [1.05, 1.74], *p* = 0.018) compared with Q2 and Q3.

The results showed that higher GV groups were associated with increased 28‐day mortality risk, except for patients with diabetes (HR [95% CI] 1.25 [0.99, 1.58], *p* = 0.063), those with AKI (HR [95% CI] 1.13 [0.97, 1.32], *p* = 0.115), and those with AG ≥ 139.7 mg/dL (HR [95% CI] 1.02 [0.89, 1.16], *p* = 0.774) (Figure 4). In terms of interaction, higher GV groups showed more significant mortality risks in subgroups of patients aged < 66 years, without diabetes, without AKI, and with AG < 139.7 mg/dL (Figures 4, S6 and S7). Kaplan–Meier analysis in each subgroup revealed significant differences in 28‐day mortality risk among different GV groups, with the GV Q4 exhibiting the highest risk (Figure S9, all *p* < 0.05).

In subgroup analysis, patients aged < 66 years, without diabetes, with AMI, and without AKI exhibited more pronounced mortality risks in the high AG and GV groups (Figure 4). Kaplan–Meier analysis showed that Group 1 had the lowest 28‐day mortality risk (Figure S10, all *p* < 0.05).

### 3.5 Association Between AG, GV, and Length of ICU Stay

**FIGURE 5:**
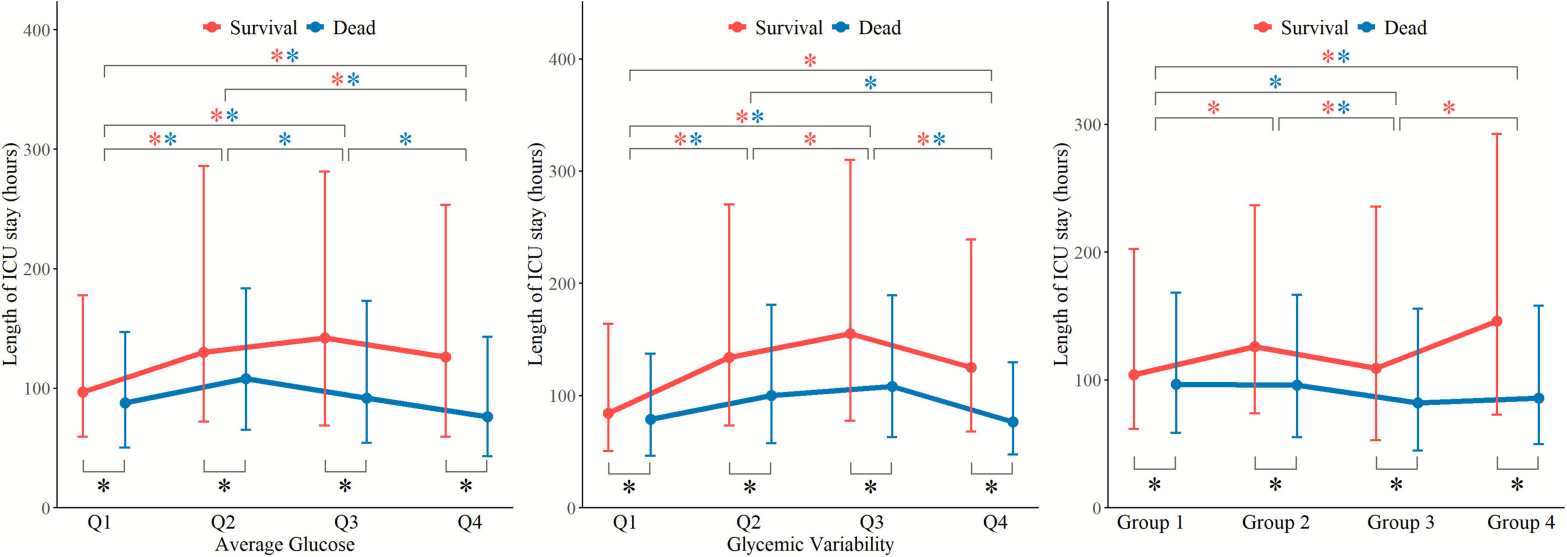
Length of ICU stay according to average glucose and glycemic variability grouping.

The length of ICU stay for patients in different groups is shown in Table S8. For AG quartile groups, Q1 had the shortest LOS among survivors, while Q4 had the shortest LOS among the dead. Additionally, LOS in survivors was significantly longer than that in nonsurvivors (Figure 5). Results from the GLM showed that after adjusting for multiple covariates, the LOS in Q1 was lower than that in Q2 and Q3, regardless of survival status (Table S9, *p* < 0.05). When analyzing AG as a continuous variable, AG was positively correlated with LOS in survivors (*ρ* = 0.0896, *p* < 0.001), but negatively correlated in nonsurvivors (*ρ* = −0.0471, *p* = 0.020). However, the GLM did not show such associations (Figure S11 and Table S9).

For GV quartile groups, Q1 had the shortest LOS among survivors, while both Q1 and Q4 had the shortest LOS among nonsurvivors. Additionally, survivors exhibited longer LOS than the dead (Figure 5). GLM results showed that the LOS in Q2 and Q3 groups was significantly longer than that in Q1, regardless of survival status (Table S9, *p* < 0.001). A positive correlation was observed between GV and LOS in survivors (*ρ* = 0.1767, *p* < 0.001), which remained significant after adjustment for multiple factors (Figure S11 and Table S9).

In the combined AG and GV groups, survivors in Group 1 and Group 3 (GV < 25.8%) had shorter LOS, whereas nonsurvivors in Group 3 and Group 4 (AG ≥ 139.7 mg/dL) exhibited shorter LOS. Notably, nonsurvivors had overall shorter LOS than survivors (Figure 5). These results were also supported by GLM analyses (Table S9, all *p* < 0.05).

## 4 Discussion

This retrospective study evaluated 6110 CA patients. The main results showed that both AG and GV during ICU hospitalization were associated with a 28‐day mortality risk. We also observed that higher AG and GV were associated with longer LOS in the ICU.

Hyperglycemia, recognized as an independent risk factor for AMI [19] and stroke [20], also correlates with poor prognosis in CA patients, as evidenced by several small‐scale trials and studies focusing on early glycemic changes [5, 14, 21]. Potential mechanisms include: Patients with poor prognosis have higher baseline blood glucose levels. Glycated hemoglobin (HbA1c), reflecting long‐term glycemic status over the past 4–12 weeks, has been independently associated with adverse neurological outcomes in the study by Lee et al. [22]. A meta‐analysis [23] further demonstrates that OHCA patients with diabetes have significantly worse in‐hospital survival and neurological outcomes, underscoring the detrimental impact of glucose metabolism disorders. In our study, diabetes was associated with higher 28‐day mortality (HR [95% CI] 1.27 [1.10, 1.48], *p* = 0.001) in patients with AG ≥ 139.7 mg/dL, whereas the opposite trend was observed in patients with lower AG (HR [95% CI] 0.69 [0.56, 0.86], *p* = 0.001), potentially due to higher mortality in diabetic patients with hypoglycemia (Figure S8). In addition, higher blood glucose levels may reflect more severe disease severity [21, 24]. Post‐CA neuroendocrine hyperactivation, immune and metabolic disorders, and organ dysfunction induce stress hyperglycemia, with its severity generally correlating with the intensity of the stress response [24]. In our study, higher AG was associated with older age, increased incidence of AMI and AKI, and higher utilization rates of MV, CRRT, and vasoactive‐inotropic medications. Finally, hyperglycemia can also exert systemic adverse effects. Hyperglycemia exacerbates immune dysregulation and inflammatory imbalance by promoting oxidative stress [25], cytokine production, and endothelial dysfunction [26, 27]. However, the latest International Consensus on Cardiopulmonary Resuscitation and Emergency Cardiovascular Care Science With Treatment Recommendations [17] does not specify a targeted glycemic range for CA patients. A randomized controlled trial [28] showed no difference in outcomes between tight glycemic control (4–6 mmol/L [72–108 mg/dL]) and moderate control (6–8 mmol/L [108–144 mg/dL]) in OHCA patients with ventricular fibrillation, consistent with our study data (HR [95% CI] 1.12 [0.94, 1.34], *p* = 0.227), which may possibly be due to the narrow glycemic range studied and 47.2% of patients having mean glucose levels outside this range. Through RCS analysis, our study determined that when AG is ≥ 139.7 mg/dL, the 28‐day mortality of patients increases significantly, while both blood glucose control ranges are near or below the “risk threshold of 139.7 mg/dL” identified in our research. Additionally, the threshold between safety and risk quantified by our study with a large sample size (*n* = 6110) provides a basis for the group design of future prospective studies, enabling more accurate verification of the effectiveness of glucose interventions. An editorial [29] proposes that for CA patients, the target should not be normal blood glucose but rather maintaining it below 10 mmol/L (180 mg/dL). European Resuscitation Council and European Society of Intensive Care Medicine guidelines 2021 [30] recommend a target glycemic range of 7.8–10.0 mmol/L (140–180 mg/dL) post‐CA. In this study, patients with an AG level < 139.7 mg/dL had a better prognosis, which may suggest the need for stricter blood glucose control after CA. Additionally, this study revealed a U‐shaped relationship between AG and prognosis, supporting the guideline recommendation of strictly avoiding hypoglycemia.

The correlation between GV and prognosis in CA patients has been confirmed in previous studies, which used the range or mean absolute glucose [31] for evaluation [5, 14, 15]. To the best of our knowledge, no study on CA has used the coefficient of variation (defined as the SD of glucose divided by the mean) as an index of GV, although this metric has been proven to correlate with mortality in various critical illnesses [11, 12, 13, 32]. High GV reflects disordered glucose regulation and, compared with sustained hyperglycemia, exacerbates oxidative stress via endothelial cell activation, leading to excessive reactive oxygen species production, inflammatory cytokine release, and enhanced apoptosis [33]. Additionally, hyperglycemia and GV disrupt immune function, inhibiting neutrophil chemotaxis and phagocytosis to increase infection risk [34, 35]. The primary causes of death after CA include poor neurological outcomes and refractory hemodynamic instability [36]. GV is an independent predictor of poor prognosis in numerous neurological disorders [13, 37], and its close association with hypoglycemia risk [38] further impairs neural recovery. Moreover, high GV affects outcomes by increasing the risk of ventricular arrhythmias [32]. Consistently, our study found that higher GV was associated with a greater prevalence of diabetes, AKI, and chronic kidney disease, as well as higher utilization rates of MV, CRRT, and vasoactive‐inotropic medications, reflecting its correlation with disease severity. A large registry study [14] involving 381 CA patients who underwent targeted temperature management (TTM) at 33°C showed that hyperglycemia was significantly associated with poor neurological prognosis (OR [95% CI]: 0.43 [0.24, 0.78], *p* = 0.006). The magnitude of blood glucose (BG) changes in patients with poor prognosis was significantly greater than that in those with good prognosis (7.1 [4.2, 11] vs. 9.6 [5.9, 13.6] mmol/L, *p* < 0.01). Although there are differences in definitions compared with our study, our research conclusions are consistent. Notably, this study did not determine the control ranges for AG and GV, which have been supplemented in our current research. A post hoc analysis of the TTM trial [5] found that patients with better prognosis had lower median blood glucose levels, while those with poorer prognosis had significantly higher GV, which is consistent with our study. Additionally, the analysis revealed that the proportion of hyperglycemia was higher in the 33°C treatment group than in the 36°C group, emphasizing the need for enhanced blood glucose monitoring during hypothermic TTM. Future studies should explore the interaction between GV and TTM and establish personalized blood glucose management strategies based on TTM protocols. Although no guidelines specify GV targets after CA, multiple studies have shown that unstable glucose control predicts poor outcomes [14, 15], consistent with our findings. Therefore, frequent blood glucose monitoring is necessary to maintain blood glucose stability during post‐CA care. Furthermore, this study demonstrates that both AG and GV correlate with LOS of the ICU.

We also conducted sensitivity analyses and subgroup analyses to enhance the robustness of outcomes. In different databases, both high AG and GV were independent predictors of 28‐day mortality in patients with CA. Although significant interactions were observed across subgroups, all demonstrated that patients with high AG and high GV had higher 28‐day mortality (Figure S7). Notably, in diabetic patients, those in AG Q1 (< 121.65 mg/dL) also exhibited poor outcomes. This may be attributed to impaired glucose regulation in diabetic patients and the significantly increased risk of hypoglycemia under strict glycemic control [39]. This highlights the need to consider diabetic comorbidity in glycemic management after CA. Additionally, significant interactions existed between different AG and GV subgroups: in subgroups with stable GV, higher AG had a greater impact on outcomes; in subgroups with lower baseline glucose, higher GV exerted a more pronounced effect. Thus, glycemic control may require simultaneous consideration of both glucose levels and variability.

This study has several limitations. First, as a retrospective study, it cannot establish a causal relationship and is inherently subject to biases. Further prospective studies and randomized controlled trials are required to validate the findings. Second, CA‐specific variables—such as the time from CA to ROSC, bystander CPR, etiologies, and TTM—and neurological outcomes were not accessible in these databases. In the present study, CA‐specific indicators and neurological function data were only supplemented and analyzed using the database of Beijing Chaoyang Hospital. The results also supported the roles of AG and GV (Tables S7–S9). However, it should be noted that the sample size for this part of the analysis was relatively small. Third, this study did not analyze the impact of TTM on blood glucose. As a key intervention regimen following CA, TTM can increase glucose levels and GV through mechanisms such as inhibiting hepatic gluconeogenesis and reducing insulin sensitivity [5, 9, 15]. Fourth, the blood glucose measurement protocols for the eICU‐CRD and MIMIC databases in this study are unknown. Differences in monitoring timing and measurement sources (including point‐of‐care capillary glucose, arterial blood gas glucose, venous biochemical glucose, etc.) may introduce measurement bias and make it difficult to meet the data consistency requirements for time‐weighted averages. Fifth, the study did not account for therapeutic interventions during ICU stay that may affect glucose levels (e.g., nutritional support). Finally, we only considered glucose levels during the ICU stay prior to the outcome, without analyzing pre‐admission glucose or glucose levels during the non‐ICU period, nor did we consider dynamic glucose changes, which limits the generalizability of the findings.

## 5 Conclusions

This study demonstrated a nonlinear relationship between AG and 28‐day mortality in CA patients, whereas GV showed a linear association. High AG and GV were independent predictors of 28‐day death risk in CA patients and were associated with a prolonged length of ICU stay.

## Author Contributions

**Zhenyu Shan:** writing – original draft, visualization, software, methodology, formal nalysis, conceptualization. **Yanrui Jia:** writing – original draft, formal analysis, data curation, conceptualization. **Xingsheng Wang:** writing – review and editing. **Guyu Zhang:** software, methodology. **Chenchen Hang:** data curation. **Le An:** data curation, conceptualization. **Rui Shao:** writing – review and editing, supervision, Conceptualization. **Ziren Tang:** writing – review and editing, supervision.

## Funding

This work was supported by High‐Level Public Health Technical Talent Building Program, Discipline Leader‐01‐01. Beijing Hospitals Authority's Ascent Plan, DFL20240302.

## Ethics Statement

This study was conducted in accordance with the guiding principles of the Helsinki Declaration. The use of eICU‐CRD (v2.0), a subset of MIMIC‐III (v1.4), and MIMIC‐IV (v3.1) databases was performed after completing the required training and obtaining relevant certifications. The use of EICU data from Beijing Chaoyang Hospital was approved by the hospital's Institutional Review Board and the Ethics Committee of Beijing Chaoyang Hospital. Due to the retrospective design, the requirement for informed consent was waived.

## Consent

The authors have nothing to report.

## Conflicts of Interest

The authors declare no conflicts of interest.

## Supporting information


**Data S1:** Supporting Information.

## Data Availability

The eICU‐CRD (v2.0), a subset of MIMIC‐III (v1.4), and MIMIC‐IV (v3.1) can be freely accessed on PhysioNet after obtaining usage permissions. Data from the EICU of Beijing Chaoyang Hospital can be requested from the corresponding authors.
