# Supplementary material for: Association of Average Glucose and Glycemic Variability With 28‐Day Mortality in Patients With Cardiac Arrest: A Retrospective Study
Source: J Diabetes. 2025 Dec 7;17(12):e70179. doi: 10.1111/1753-0407.70179 (PMC12682468; doi:10.1111/1753-0407.70179)
Supplement: Supplementary file 1 — Data S1: Supporting Information. [file JDB-17-e70179-s001.docx]

Table S1. Baseline characteristics of patients with cardiac arrest according to average glucose.

| **Variable** | **Overall (n=6110)** | **Q 1 (n=1528)** | **Q 2 (n=1527)** | **Q 3 (n=1527)** | **Q 4 (n=1528)** | **P-value** |
| --- | --- | --- | --- | --- | --- | --- |
| **Demographics** |  |  |  |  |  |  |
| Female, n (%) | 2421 (39.62 %) | 561 (36.71 %) | 599 (39.23 %) | 612 (40.08 %) | 649 (42.47 %) | 0.008 |
| Age, year | 66 (55, 76) | 62 (50, 74) | 66 (54, 77) | 68 (58, 76) | 67 (57, 76) | <0.001 |
| Race, n (%) |  |  |  |  |  | <0.001 |
| White | 3997 (65.42 %) | 1017 (66.56 %) | 1015 (66.47 %) | 1036 (67.85 %) | 929 (60.8 %) |  |
| Black | 788 (12.9 %) | 215 (14.07 %) | 192 (12.57 %) | 190 (12.44 %) | 191 (12.5 %) |  |
| Other/Unknown | 1325 (21.69 %) | 296 (19.37 %) | 320 (20.96 %) | 301 (19.71 %) | 408 (26.7 %) |  |
| BMI, kg/m^2^ | 28.04 (24.24, 33.54) | 26.6 (23.04, 31.41) | 27.62 (23.94, 33.3) | 28.52 (24.8, 33.92) | 29.64 (25.21, 35.7) | <0.001 |
| **Vital signs** |  |  |  |  |  |  |
| MAP, mmHg | 82 (69, 98) | 83 (70, 99) | 83 (69, 98) | 82 (69, 99) | 82 (68, 97) | 0.254 |
| Temperature, ℃ | 36.5 (35.6, 36.9) | 36.5 (35.7, 36.9) | 36.5 (35.6, 37.0) | 36.5 (35.6, 36.9) | 36.5 (35.5, 36.9) | 0.344 |
| Heart rate, bpm | 89 (75, 106) | 88 (73, 105) | 88 (75, 105) | 88 (75, 106) | 92 (76, 109) | <0.001 |
| Respiratory rate, bpm | 20 (16, 24) | 19 (16, 24) | 19 (16, 24) | 20 (16, 24) | 20 (16, 25) | <0.001 |
| **Comorbidities** |  |  |  |  |  |  |
| Hypertension, n (%) | 5238 (85.73 %) | 1290 (84.42 %) | 1298 (85 %) | 1335 (87.43 %) | 1315 (86.06 %) | 0.085 |
| Diabetes, n (%) | 1454 (23.8 %) | 154 (10.08 %) | 246 (16.11 %) | 381 (24.95 %) | 673 (44.04 %) | <0.001 |
| Cerebral infarction, n (%) | 219 (3.58 %) | 38 (2.49 %) | 40 (2.62 %) | 54 (3.54 %) | 87 (5.69 %) | <0.001 |
| Acute myocardial infarction, n (%) | 1071 (17.53 %) | 232 (15.18 %) | 265 (17.35 %) | 270 (17.68 %) | 304 (19.9 %) | 0.008 |
| Acute kidney injury, n (%) | 2051 (33.57 %) | 417 (27.29 %) | 469 (30.71 %) | 517 (33.86 %) | 648 (42.41 %) | <0.001 |
| Liver cirrhosis, n (%) | 157 (2.57 %) | 47 (3.08 %) | 42 (2.75 %) | 31 (2.03 %) | 37 (2.42 %) | 0.304 |
| Chronic kidney disease, n (%) | 1178 (19.28 %) | 308 (20.16 %) | 268 (17.55 %) | 267 (17.49 %) | 335 (21.92 %) | 0.004 |
| Malignant tumor, n (%) | 419 (6.86 %) | 105 (6.87 %) | 100 (6.55 %) | 113 (7.4 %) | 101 (6.61 %) | 0.771 |
| **Laboratory measurements** |  |  |  |  |  |  |
| WBC counts, K/uL | 13.4 (9.6, 19.12) | 11.8 (8.6, 16.8) | 13.3 (9.78, 18.8) | 13.8 (9.9, 19.6) | 14.6 (10.38, 21) | <0.001 |
| Hemoglobin, g/dL | 11 (9.3, 13.3) | 11.1 (9.3, 13.2) | 11 (9.3, 13.2) | 11.2 (9.4, 13.2) | 10.9 (9.3, 13.6) | 0.473 |
| Platelet counts, K/μL | 195 (145, 260) | 187 (139, 252) | 191 (146, 255) | 202 (151, 262) | 203 (147, 267.5) | <0.001 |
| BUN, mg/dL | 23 (16, 39) | 20 (14, 35.5) | 21 (15, 35) | 24 (18, 38) | 29 (20, 47) | <0.001 |
| Creatinine, mg/dL | 1.2 (0.9, 2.12) | 1.1 (0.8, 2.13) | 1.1 (0.86, 1.9) | 1.23 (0.96, 2.1) | 1.5 (1.09, 2.38) | <0.001 |
| Total calcium, mg/dL | 8.2 (7.6, 8.7) | 8.2 (7.6, 8.7) | 8.1 (7.6, 8.7) | 8.1 (7.6, 8.7) | 8.2 (7.6, 8.7) | 0.275 |
| Sodium, mmol/L | 139 (136, 142) | 139 (136, 142) | 139 (136, 142) | 139 (135, 142) | 138 (135, 142) | <0.001 |
| Potassium, mmol/L | 4.1 (3.7, 4.7) | 4.1 (3.68, 4.6) | 4.1 (3.7, 4.6) | 4.1 (3.6, 4.8) | 4.2 (3.7, 4.9) | <0.001 |
| Chloride, mmol/L | 104 (100, 108.13) | 105 (100, 109) | 105 (101, 109) | 104 (100, 108.4) | 103 (98, 107) | <0.001 |
| Anion gap, mmol/L | 14 (11, 18) | 13 (10, 17) | 14 (11, 17) | 14 (11, 18) | 16 (12, 20) | <0.001 |
| Average glucose, mg/dL | 139.93 (121.65, 171.74) | 110.46 (101.7, 116.51) | 131.5 (126.72, 136.31) | 154.36 (147.8, 162.25) | 195.56 (182.64, 225.16) | <0.001 |
| SD of glucose, mg/dL | 36.13 (24.8, 57.49) | 22.37 (16.34, 31.08) | 30.91 (23.7, 42.02) | 44.79 (32.34, 58.86) | 65.11 (48.38, 90.49) | <0.001 |
| Glycemic variability, % | 25.84 (18.89, 35.8) | 20.59 (15.25, 28.69) | 23.4 (17.97, 31.65) | 28.77 (21, 37.78) | 31.76 (24.1, 42.67) | <0.001 |
| **Treatments** |  |  |  |  |  |  |
| Mechanical ventilation, n (%) | 4491 (73.5 %) | 1041 (68.13 %) | 1117 (73.15 %) | 1160 (75.97 %) | 1173 (76.77 %) | <0.001 |
| CRRT, n (%) | 686 (11.23 %) | 157 (10.27 %) | 150 (9.82 %) | 180 (11.79 %) | 199 (13.02 %) | 0.022 |
| Vasoactive-inotropic drugs, n (%) | 4315 (70.62 %) | 988 (64.66 %) | 1094 (71.64 %) | 1090 (71.38 %) | 1143 (74.8 %) | <0.001 |
| Insulin, n (%) | 4016 (65.73 %) | 789 5(1.64 %) | 993 (65.03 %) | 1087 (71.19 %) | 1147 (75.07 %) | <0.001 |
| **Outcomes** |  |  |  |  |  |  |
| 28-day mortality, n (%) | 2782 (45.53 %) | 522 (34.16 %) | 619 (40.54 %) | 762 (49.9 %) | 879 (57.53 %) | <0.001 |
| Length of ICU stay, hour | 102.26 (57.96, 194.67) | 93.22 (55.47, 166.81) | 117.70 (68.78, 235.34) | 114.07 (60.73, 213.43) | 90.07 (48.08, 186.36) | <0.001 |

Data are shown as median (IQR) or n (%).

Average glucose Q1: AG <121.65mg/dL, Q2: 121.65mg/dL≤ AG <141.59mg/dL, Q3: 141.59mg/dL≤ AG <171.75mg/dL, Q4: AG ≥171.75mg/dL.

P-value indicates the chi-square test or Kruskal-Wallis test.

BMI: body mass index, MAP: mean arterial pressure, WBC: white blood cell, BUN: blood urea nitrogen, CRRT: continuous renal replacement therapy, AG: average glucose, SD: standard deviation, ICU: Intensive Care Unit.

Table S2. Baseline characteristics of patients with cardiac arrest according to glycemic variability.

| **Variable** | **Overall (n=6110)** | **Q 1 (n=1528)** | **Q 2 (n=1527)** | **Q 3 (n=1527)** | **Q 4 (n=1528)** | **P-value** |
| --- | --- | --- | --- | --- | --- | --- |
| **Demographics** |  |  |  |  |  |  |
| Female, n (%) | 2421 (39.62 %) | 537 (35.14 %) | 573 (37.52 %) | 638 (41.78 %) | 673 (44.04 %) | <0.001 |
| Age, year | 66 (55, 76) | 66 (53, 76) | 66 (54, 77) | 67 (56, 76) | 66 (55, 75) | 0.303 |
| Race, n (%) |  |  |  |  |  |  |
| White | 3997 (65.42 %) | 1073 (70.22 %) | 1009 (66.08 %) | 947 (62.02 %) | 968 (63.35 %) | <0.001 |
| Black | 788 (12.9 %) | 165 (10.8 %) | 169 (11.07 %) | 214 (14.01 %) | 240 (15.71 %) | <0.001 |
| Other/Unknown | 1325 (21.69 %) | 290 (18.98 %) | 349 (22.86 %) | 366 (23.97 %) | 320 (20.94 %) | <0.001 |
| BMI, kg/m^2^ | 28.04 (24.24, 33.54) | 28.18 (24.21, 33.33) | 28.37 (24.57, 33.81) | 27.73 (24.36, 33.64) | 27.8 (23.82, 33.67) | 0.276 |
| **Vital signs** |  |  |  |  |  |  |
| MAP, mmHg | 82 (69, 98) | 83 (71, 98) | 82 (69, 98) | 83 (70, 98) | 81 (67, 97) | 0.067 |
| Temperature, ℃ | 36.5 (35.56, 36.94) | 36.61 (35.9, 37) | 36.5 (35.7, 37) | 36.44 (35.44, 36.9) | 36.33 (35, 36.83) | <0.001 |
| Heart rate, bpm | 89 (75, 106) | 87 (73, 103) | 89 (75, 106) | 90 (75, 107) | 90 (76, 111) | <0.001 |
| Respiratory rate, bpm | 20 (16, 24) | 19 (16, 24) | 19 (16, 24) | 20 (16, 24) | 20 (16, 25) | <0.001 |
| **Comorbidities** |  |  |  |  |  |  |
| Hypertension, n (%) | 5238 (85.73 %) | 1309 (85.67 %) | 1318 (86.31 %) | 1279 (83.76 %) | 1332 (87.17 %) | 0.045 |
| Diabetes, n (%) | 1454 (23.8 %) | 197 (12.89 %) | 294 (19.25 %) | 447 (29.27 %) | 516 (33.77 %) | <0.001 |
| Cerebral infarction, n (%) | 219 (3.58 %) | 41 (2.68 %) | 53 (3.47 %) | 72 (4.72 %) | 53 (3.47 %) | 0.026 |
| Acute myocardial infarction, n (%) | 1071 (17.53 %) | 256 (16.75 %) | 266 (17.42 %) | 268 (17.55 %) | 281 (18.39 %) | 0.698 |
| Acute kidney injury, n (%) | 2051 (33.57 %) | 393 (25.72 %) | 485 (31.76 %) | 560 (36.67 %) | 613 (40.12 %) | <0.001 |
| Liver cirrhosis, n (%) | 157 (2.57 %) | 44 (2.88 %) | 41 (2.69 %) | 35 (2.29 %) | 37 (2.42 %) | 0.748 |
| Chronic kidney disease, n (%) | 1178 (19.28 %) | 218 (14.27 %) | 283 (18.53 %) | 320 (20.96 %) | 357 (23.36 %) | <0.001 |
| Malignant tumor, n (%) | 419 (6.86 %) | 116 (7.59 %) | 115 (7.53 %) | 90 (5.89 %) | 98 (6.41 %) | 0.167 |
| **Laboratory measurements** |  |  |  |  |  |  |
| WBC counts, K/uL | 13.4 (9.6, 19.12) | 12.4 (9.3, 17.2) | 13.1 (9.4, 18.54) | 13.9 (9.7, 19.6) | 14.6 (10.2, 21.1) | <0.001 |
| Hemoglobin, g/dL | 11 (9.3, 13.3) | 11.4 (9.55, 13.3) | 11 (9.4, 13.5) | 10.9 (9.2, 13.3) | 10.9 (9.1, 13.1) | <0.001 |
| Platelet counts, K/μL | 195 (145, 260) | 194 (149, 256) | 192 (143, 255) | 193 (142, 260.5) | 202 (147, 267) | 0.062 |
| BUN, mg/dL | 23 (16, 39) | 20 (14, 33) | 22 (15.13, 37) | 24 (17, 42) | 27 (19, 45) | <0.001 |
| Creatinine, mg/dL | 1.2 (0.9, 2.12) | 1.03 (0.8, 1.7) | 1.13 (0.86, 1.96) | 1.3 (1, 2.26) | 1.5 (1.1, 2.56) | <0.001 |
| Total calcium, mg/dL | 8.2 (7.6, 8.7) | 8.2 (7.7, 8.7) | 8.1 (7.6, 8.7) | 8.2 (7.6, 8.7) | 8.1 (7.5, 8.6) | <0.001 |
| Sodium, mmol/L | 139 (136, 142) | 139 (136, 142) | 139 (136, 142) | 139 (136, 142) | 138 (135, 142) | <0.001 |
| Potassium, mmol/L | 4.1 (3.7, 4.7) | 4.1 (3.7, 4.57) | 4.1 (3.7, 4.7) | 4.1 (3.6, 4.7) | 4.2 (3.6, 4.9) | 0.002 |
| Chloride, mmol/L | 104 (100, 108.13) | 105 (101, 108) | 105 (101, 109) | 104 (100, 109) | 103 (98, 108) | <0.001 |
| Anion gap, mmol/L | 14 (11, 18) | 13 (10, 16) | 13 (10, 17) | 14.2 (11.1, 18) | 16 (13, 20) | <0.001 |
| Average glucose, mg/dL | 139.93 (121.65, 171.74) | 126.02 (111.98, 148.09) | 134 (119.98, 160.26) | 145.42 (126.08, 175.38) | 160.31 (137.81, 191.84) | <0.001 |
| SD of glucose, mg/dL | 36.13 (24.8, 57.49) | 18.55 (14.38, 23.03) | 30.2 (26.36, 36.61) | 45.05 (37.8, 54.74) | 71.61 (58.94, 93.38) | <0.001 |
| Glycemic variability, % | 25.84 (18.89, 35.8) | 14.9 (11.75, 16.97) | 22.42 (20.62, 24.3) | 30.53 (28.29, 32.86) | 43.31 (39.4, 51.51) | <0.001 |
| **Treatments** |  |  |  |  |  |  |
| Mechanical ventilation, n (%) | 4491 (73.5 %) | 1023 (66.95 %) | 1147 (75.11 %) | 1169 (76.56 %) | 1152 (75.39 %) | <0.001 |
| CRRT, n (%) | 686 (11.23 %) | 94 (6.15 %) | 172 (11.26 %) | 199 (13.03 %) | 221 (14.46 %) | <0.001 |
| Vasoactive-inotropic drugs, n (%) | 4315 (70.62 %) | 922 (60.34 %) | 1069 (70.01 %) | 1126 (73.74 %) | 1198 (78.4 %) | <0.001 |
| Insulin, n (%) | 4016 (65.73 %) | 816 (53.4 %) | 992 (64.96 %) | 1074 (70.33 %) | 1134 (74.21 %) | <0.001 |
| **Outcomes** |  |  |  |  |  |  |
| 28-day mortality, n (%) | 2782 (45.53 %) | 560 (36.65 %) | 637 (41.72 %) | 718 (47.02 %) | 867 (56.74 %) | <0.001 |
| Length of ICU stay, hour | 102.26 (57.96, 194.67) | 82.39 (48.91, 155.91) | 119.28 (67.23, 230.84) | 125.35 (68.56, 233.56) | 92.51 (54.69, 174.28) | <0.001 |

Data are shown as median (IQR) or n (%).

Glycemic variability Q1: GV <18.86%, Q2: 18.86%≤ GV <26.21%, Q3: 26.21%≤ GV <35.8%, Q4: GV ≥35.8%.

P-value indicates the chi-square test or Kruskal-Wallis test.

BMI: body mass index, MAP: mean arterial pressure, WBC: white blood cell, BUN: blood urea nitrogen, CRRT: continuous renal replacement therapy, GV: glycemic variability, SD: standard deviation, ICU: Intensive Care Unit.

Table S3. Restricted Cubic Spline and Cox proportional hazards models analyses for AG, GV and 28-day mortality in cardiac arrest patients.

|  | Model 1 | | | Model 2 | | | Model 3 | | | Model 4 | | | Model 5 | | | Model 6 | | |
| --- | --- | --- | --- | --- | --- | --- | --- | --- | --- | --- | --- | --- | --- | --- | --- | --- | --- | --- |
| **RCS** | knot | P for all | P for Nonlinear | knot | P for all | P for Nonlinear | knot | P for all | P for Nonlinear | knot | P for all | P for Nonlinear | knot | P for all | P for Nonlinear | knot | P for all | P for Nonlinear |
| AG | 5 | <0.001 | <0.001 | 5 | <0.001 | <0.001 | 5 | <0.001 | <0.001 | 5 | <0.001 | <0.001 | 5 | <0.001 | <0.001 | 5 | <0.001 | <0.001 |
| GV | 3 | <0.001 | 0.5841 | 3 | <0.001 | 0.43 | 3 | <0.001 | 0.345 | 3 | <0.001 | 0.1916 | 3 | <0.001 | 0.2243 | 3 | <0.001 | 0.0576 |
|  | | | | | | | | | | | | | | | | | | |
|  | Model 1 | | | Model 2 | | | Model 3 | | | Model 4 | | | Model 5 | | | Model 6 | | |
| **Cox** | HR (95%CI) | | P-value | HR (95%CI) | | P-value | HR (95%CI) | | P-value | HR (95%CI) | | P-value | HR (95%CI) | | P-value | HR (95%CI) | | P-value |
| ln (AG) | 1.69 (1.58, 1.81) | | <0.001 | 1.69 (1.58, 1.81) | | <0.001 | 1.66 (1.54, 1.78) | | <0.001 | 1.56 (1.45, 1.67) | | <0.001 | 1.52 (1.41, 1.64) | | <0.001 | 1.53 (1.42, 1.66) | | <0.001 |
| ln (GV) | 1.60 (1.49, 1.72) | | <0.001 | 1.60 (1.48, 1.72) | | <0.001 | 1.55 (1.43, 1.67) | | <0.001 | 1.51 (1.40, 1.63) | | <0.001 | 1.39 (1.28, 1.50) | | <0.001 | 1.33 (1.23, 1.45) | | <0.001 |

HR means each unit increment in ln (AG) or ln (GV).

Model 1: unadjusted.

Model 2: Model 1 + Gender, Age, Race, and BMI.

Model 3: Model 2 + MAP, Temperature, Heart rate, and Respiratory rate.

Model 4: Model 3 + Hypertension, Diabetes, Cerebral infarction, Acute myocardial infarction, Acute kidney injury, Liver cirrhosis, Chronic kidney disease, and Malignant tumor.

Model 5: Model 4 + WBC counts, Hemoglobin, Platelet counts, BUN, Creatinine, Total calcium, Sodium, Potassium, Chloride, and Anion gap.

Model 6: Model 5 + Mechanical ventilation, CRRT, Vasoactive-inotropic drugs, and Insulin.

RCS: Restricted Cubic Spline, HR: hazard ratio, BMI: body mass index, MAP: mean arterial pressure, WBC: white blood cell, BUN: blood urea nitrogen, CRRT: continuous renal replacement therapy, AG: average glucose, GV: glycemic variability.

Table S4. Cox proportional hazards models analyses for different groups and 28-day mortality in different databases.

|  | **eICU-CRD Model** | |  | **MIMIC Model** | | |
| --- | --- | --- | --- | --- | --- | --- |
|  | **HR (95%CI)** | **P** | **P**-Adjusted | **HR (95%CI)** | **P** | **P**-Adjusted |
| **Average glucose** | | | |  | | |
| Continuous | 1.006 (1.005, 1.007) | **<0.001** | **<0.001** | 1.00 (1.000, 1.000) | 0.729 | 1 |
| Q1 | 1 | - |  | 1 | - |  |
| Q2 | 1.13 (0.97, 1.32) | 0.117 | 1 | 1.00 (0.83, 1.20) | 0.989 | 1 |
| Q3 | 1.35 (1.15, 1.57) | **<0.001** | **0.005** | 1.50 (1.25, 1.80) | **<0.001** | **<0.001** |
| Q4 | 1.99 (1.70, 2.33) | **<0.001** | **<0.001** | 1.66 (1.25, 2.03) | **<0.001** | **<0.001** |
| **Glycemic variability** | | | |  | | |
| Continuous | 1.012 (1.009, 1.016 | **<0.001** | **<0.001** | 1.00 (0.999, 1.001) | 0.782 | **1** |
| Q1 | 1 | - |  | 1 | - |  |
| Q2 | 1.10 (0.94, 1.28) | 0.228 | 1 | 0.93 (0.77, 1.12) | 0.457 | 1 |
| Q3 | 1.10 (0.95, 1.28) | 0.219 | 1 | 1.02 (0.85, 1.22) | 0.826 | 1 |
| Q4 | 1.51 (1.30, 1.75) | **<0.001** | **<0.001** | 1.20 (1.00, 1.45) | 0.056 | 1 |
| **Average glucose and Glycemic variability** | | | |  | | |
| Group 1 | 1 | - |  | 1 | - |  |
| Group 2 | 1.35 (1.15, 1.58) | **<0.001** | **0.008** | 1.32 (1.08, 1.60) | **0.006** | 0.191 |
| Group 3 | 1.73 (1.48, 2.02) | **<0.001** | **<0.001** | 1.94 (1.59, 2.36) | **<0.001** | **<0.001** |
| Group 4 | 1.72 (1.49, 1.98) | **<0.001** | **<0.001** | 1.67 (1.39, 2.00) | **<0.001** | **<0.001** |

Average glucose Q1: AG <121.65mg/dL, Q2: 121.65mg/dL≤ AG <141.59mg/dL, Q3: 141.59mg/dL≤ AG <171.75mg/dL, Q4: AG ≥171.75mg/dL.

Glycemic variability Q1: GV <18.86%, Q2: 18.86%≤ GV <26.21%, Q3: 26.21%≤ GV <35.8%, Q4: GV ≥35.8%.

Group 1: AG <139.7mg/dL and GV <25.8%; Group 2: AG <139.7mg/dL and GV ≥25.8%; Group 3: AG ≥139.7mg/dL and GV <25.8%; Group 4: AG ≥139.7mg/dL and GV ≥25.8%.

Model: adjusted for Gender, Age, Race, BMI, MAP, Temperature, Heart rate, Respiratory rate, Hypertension, Diabetes, Cerebral infarction, Acute myocardial infarction, Acute kidney injury, Liver cirrhosis, Chronic kidney disease, Malignant tumor, WBC counts, Hemoglobin, Platelet counts, BUN, Creatinine, Total calcium, Sodium, Potassium, Chloride, Anion gap, Mechanical ventilation, CRRT, Vasoactive-inotropic drugs, and Insulin.

Table S5. CA-specific variables of CA patients according to average glucose and glycemic variability in Beijing Chaoyang hospital.

| Group by **Average glucose** | | | | | |
| --- | --- | --- | --- | --- | --- |
| Variable | Q1 (n=10) | Q2 (n=19) | Q3 (n=23) | Q4 (n=101) | P-value |
| TROSC, min | 14.5 (3, 39) | 20 (16, 27.5) | 15 (9, 34.5) | 31 (15, 60) | **0.038** |
| Cardiac, n | 3 (37.5%) | 10 (52.6%) | 13 (56.5%) | 56 (55.4%) | 0.808 |
| Bystander CPR, n | 6 (75%) | 15 (78.9%) | 16 (69.6%) | 71 (70.3%) | 0.939 |
| Shockable rhythm, n | 2 (25%) | 8 (42.1%) | 3 (13%) | 25 (24.8%) | 0.377 |
| 33℃ TTM, n | 5 (50%) | 12 (63.2%) | 8 (34.8%) | 58 (57.4%) | 0.219 |
| 28-day mortality, n | 2 (25%) | 4 (21.1%) | 15 (65.2%) | 60 (59.4%) | **0.003** |
| Poor neurological outcomes, n | 7 (70%) | 9 (50%) | 18 (78.6%) | 85 (84.2%) | **0.006** |
| Group by **Glycemic variability** | | | | | |
| Variable | Q1 (n=18) | Q2 (n=47) | Q3 (n=63) | Q4 (n=23) | P-value |
| TROSC, min | 35.5 (16.25, 72) | 21 (14, 46.5) | 20 (12, 42) | 32 (14.5, 53) | 0.463 |
| Cardiac, n | 14 (77.8%) | 24 (51.1%) | 30 (47.6%) | 14 (60.9%) | 0.124 |
| Bystander CPR, n | 14 (77.8%) | 36 (76.6%) | 48 (76.2%) | 10 (43.5%) | **0.023** |
| Shockable rhythm, n | 11 (61.1%) | 13 (27.7%) | 12 (19%) | 2 (8.7%) | **0.009** |
| 33℃ TTM, n | 9 (50%) | 28 (59.6%) | 33 (52.4%) | 12 (52.2%) | 0.708 |
| 28-day mortality, n | 11 (61.1%) | 26 (55.3%) | 29 (46%) | 4 (17.4%) | **0.011** |
| Poor neurological outcomes, n | 13 (72.2%) | 30 (66.7%) | 51 (81.0%) | 23 (100%) | **0.004** |
| Group by **Average glucose and Glycemic variability** | | | | | |
| Variable | Q1 (n=19) | Q2 (n=8) | Q3 (n=42) | Q4 (n=82) | P-value |
| TROSC, min | 20 (11, 31) | 20.5 (13, 36) | 30.5 (15.25, 69) | 25 (11.25, 49) | 0.198 |
| Cardiac, n | 11 (57.9%) | 2 (25%) | 27 (64.3%) | 42 (51.2%) | 0.187 |
| Bystander CPR, n | 14 (73.7%) | 7 (87.5%) | 35 (83.3%) | 52 (63.4%) | 0.091 |
| Shockable rhythm, n | 10 (52.6%) | 4 (50%) | 14 (33.3%) | 14 (17.1%) | **0.005** |
| 33℃ TTM, n | 10 (52.6%) | 6 (75%) | 26 (61.9%) | 41 (50%) | 0.391 |
| 28-day mortality, n | 3 (15.8%) | 3 (37.5%) | 22 (52.4%) | 53 (64.6%) | **<0.001** |
| Poor neurological outcomes, n | 10 (52.6%) | 5 (62.5%) | 31 (73.8%) | 74 (90.2%) | **<0.001** |

TROSC: Time to ROSC, CPR: Cardio Pulmonary Resuscitation.

P-value indicates the chi-square test or Kruskal-Wallis test.

Table S6. Cox proportional hazards models analyses for different groups and 28-day mortality in CA patients.

|  | **Model 1** | | |  | **Model 2** | | |
| --- | --- | --- | --- | --- | --- | --- | --- |
|  | **HR (95%CI)** | **P** | **P**-Adjusted | | **HR (95%CI)** | **P** | **P**-Adjusted |
| **Average glucose** | | | | |  | | |
| Continuous | 1.006 (1.002, 1.010) | **0.006** | **0.005** | | 1.004 (0.998, 1.011) | 0.206 | 1 |
| Q1 | 1 | - |  | | 1 | - |  |
| Q2 | 0.760 (0.14, 4.15) | 0.752 | 1 | | 0.36 (0.05, 2.63) | 0.313 | 1 |
| Q3 | 3.06 (0.70, 13.4) | 0.137 | 0.412 | | 2.63 (0.51, 13.6) | 0.249 | 1 |
| Q4 | 3.02 (0.74, 12.38) | 0.124 | 0.371 | | 2.40 (0.49, 11.6) | 0.275 | 1 |
| **Glycemic variability** | | | | |  | | |
| Continuous | 501.1 (32.73, 7671) | **<0.001** | **<0.001** | | 118.5 (47.7, 2945) | **<0.001** | **<0.001** |
| Q1 | 1 | - |  | | 1 | - |  |
| Q2 | 1.17 (0.48, 2.63) | 0.799 | 1 | | 1.79 (0.60, 5.34) | 0.297 | 1 |
| Q3 | 1.38 (0.61, 3.11) | 0.442 | 1 | | 2.32 (0.81, 6.64) | 0.117 | 1 |
| Q4 | 3.31 (1.39, 7.91) | **0.007** | **0.021** | | 5.96 (1.84, 19.3) | **0.003** | **0.091** |
| **Average glucose and Glycemic variability** | | | | |  | | |
| Group 1 | 1 | - |  | | 1 | - |  |
| Group 2 | 2.68 (0.54, 13.3) | 0.419 | 0.684 | | 9.24 (1.13, 75.3) | 0.038 | 1 |
| Group 3 | 4.47 (1.33, 14.9) | **0.015** | **0.046** | | 9.54 (1.84, 49.3) | **0.007** | 0.229 |
| Group 4 | 5.84 (1.82, 18.7) | **0.003** | **0.009** | | 13.6 (2.84, 65.0) | **0.001** | **0.034** |

Average glucose Q1: AG <121.65mg/dL, Q2: 121.65mg/dL≤ AG <141.59mg/dL, Q3: 141.59mg/dL≤ AG <171.75mg/dL, Q4: AG ≥171.75mg/dL.

Glycemic variability Q1: GV <18.86%, Q2: 18.86%≤ GV <26.21%, Q3: 26.21%≤ GV <35.8%, Q4: GV ≥35.8%.

Group 1: AG <139.7mg/dL and GV <25.8%; Group 2: AG <139.7mg/dL and GV ≥25.8%; Group 3: AG ≥139.7mg/dL and GV <25.8%; Group 4: AG ≥139.7mg/dL and GV ≥25.8%.

Model 1: unadjusted. Model 2: Model 1 +TROSC, Cardiac, Bystander CPR, Shockable rhythm, Gender, Age, Race, BMI, MAP, Temperature, Heart rate, Respiratory rate, Hypertension, Diabetes, Cerebral infarction, Acute myocardial infarction, Acute kidney injury, Liver cirrhosis, Chronic kidney disease, Malignant tumor, WBC counts, Hemoglobin, Platelet counts, BUN, Creatinine, Total calcium, Sodium, Potassium, Chloride, Anion gap, TTM, Mechanical ventilation, CRRT, Vasoactive-inotropic drugs, and Insulin.

Table S7. Logistic regression models analyses for different groups and neurological outcomes in CA patients.

|  | **Model 1** | |  | **Model 2** | |  |
| --- | --- | --- | --- | --- | --- | --- |
|  | **OR (95%CI)** | **P** | **P**-Adjusted | **OR (95%CI)** | **P** | **P**-Adjusted |
| **Average glucose** | | | | | | |
| Continuous | 1.01 (1.00, 1.02) | **0.004** | **0.008** | 1.03 (1.00, 1.06) | **0.033** | **1** |
| Q1 | 1 | - | - | 1 | - | - |
| Q2 | 0.67 (0.11, 3.54) | 0.638 | 1 | 0.59 (0.09, 3.66) | 0.571 | 1 |
| Q3 | 2.85 (0.44, 17.7) | 0.252 | 1 | 2.67 (0.55, 13.09) | 0.226 | 1 |
| Q4 | 3.44 (0.65, 15.6) | 0.114 | 0.457 | 2.05 (0.43, 9.75) | 0.368 | 1 |
| **Glycemic variability** | | | | | | |
| Continuous | 4373 (20.5, 1775714) | **0.004** | **0.007** | 15528 (0.09, 4.9*10^10) | 0.142 | 1 |
| Q1 | 1 | - | - | 1 | - |  |
| Q2 | 0.82 (0.23, 2.63) | 0.747 | 1 | 0.59 (0.002, 239) | 0.852 | 1 |
| Q3 | 1.82 (0.05, 6.00) | 0.337 | 1 | 82 (0.60, 294246) | 0.157 | 1 |
| Q4 | 4*10^8 (0, 3*10^177) | 0.990 | 1 | 13.4 (0.08, 14434) | 0.386 | 1 |
| **Average glucose and Glycemic variability** | | | | | | |
| Group 1 | 1 | - | - | 1 | - | - |
| Group 2 | 1.50 (0.45, 2.80) | 0.638 | 1 | 0.48 (0.003, 48.2) | 0.203 | 1 |
| Group 3 | 2.54 (0.82, 8.03) | 0.107 | 0.430 | 22.7 (1.16, 1193) | 0.065 | 1 |
| Group 4 | 8.32 (2.64, 27.5) | **<0.001** | **0.001** | 28.4 (1.78, 854) | **0.028** | 0.906 |

Average glucose Q1: AG <121.65mg/dL, Q2: 121.65mg/dL≤ AG <141.59mg/dL, Q3: 141.59mg/dL≤ AG <171.75mg/dL, Q4: AG ≥171.75mg/dL.

Glycemic variability Q1: GV <18.86%, Q2: 18.86%≤ GV <26.21%, Q3: 26.21%≤ GV <35.8%, Q4: GV ≥35.8%.

Group 1: AG <139.7mg/dL and GV <25.8%; Group 2: AG <139.7mg/dL and GV ≥25.8%; Group 3: AG ≥139.7mg/dL and GV <25.8%; Group 4: AG ≥139.7mg/dL and GV ≥25.8%.

Model 1: unadjusted. Model 2: Model 1 +TROSC, Cardiac, Bystander CPR, Shockable rhythm, Gender, Age, Race, BMI, MAP, Temperature, Heart rate, Respiratory rate, Hypertension, Diabetes, Cerebral infarction, Acute myocardial infarction, Acute kidney injury, Liver cirrhosis, Chronic kidney disease, Malignant tumor, WBC counts, Hemoglobin, Platelet counts, BUN, Creatinine, Total calcium, Sodium, Potassium, Chloride, Anion gap, TTM, Mechanical ventilation, CRRT, Vasoactive-inotropic drugs, and Insulin.

Table S8. The differences in the length of ICU stay among patients in different groups and with different prognoses.

| **Average glucose** | | | | | | | | | | | |
| --- | --- | --- | --- | --- | --- | --- | --- | --- | --- | --- | --- |
|  | Overall |  | Q1 |  | Q2 |  | Q3 |  | Q4 |  | P |
| Overall | 102.26 (57.96, 194.67) | | 93.22 (55.47, 166.81) | | 117.70 (68.78, 235.34) | | 114.07 (60.73, 213.43) | | 90.07 (48.08, 186.36) | | <0.001 |
| Survival | 119.53 (64.75, 238.19) | | 96.84 (59.47, 177.81) | | 130.49 (71.90, 285.95) | | 141.53 (68.92, 281.34) | | 125.57 (59.33, 253.48) | | <0.001 |
| Dead | 89.00 (50.64, 162.77) | | 87.78 (50.43, 147.08) | | 108.03 (65.46, 183.69) | | 91.64 (54.23, 173.15) | | 76.15 (43.21, 143.00) | | <0.001 |
| **Glycemic variability** | | | | | | | | | | | |
|  | Overall |  | Q1 |  | Q2 |  | Q3 |  | Q4 |  | P |
| Overall | 102.26 (57.96, 194.67) | | 82.39 (48.91, 155.91) | | 119.28 (67.23, 230.84) | | 125.35 (68.56, 233.56) | | 92.51 (54.69, 174.28) | | <0.001 |
| Survival | 119.53 (64.75, 238.19) | | 84.04 (50.24, 164.06) | | 134.08 (73.47, 270.27) | | 155.42 (77.52, 309.99) | | 124.96 (68.03, 239.07) | | <0.001 |
| Dead | 89.00 (50.64, 162.77) | | 78.77 (46.38, 137.45) | | 100.16 (57.60, 180.77) | | 108.28 (62.75, 189.37) | | 76.39 (47.51, 129.56) | | <0.001 |
| **Average glucose and Glycemic variability** | | | | | | | | | | | |
|  | Overall |  | Group 1 |  | Group 2 |  | Group 3 |  | Group 4 |  | P |
| Overall | 102.26 (57.96, 194.67) | | 100.65 (60.16, 191,94) | | 100.02 (65.26, 194.12) | | 89.21 (47.97, 186.32) | | 106.99 (59.59, 207.09) | | <0.001 |
| Survival | 119.53 (64.75, 238.19) | | 103.52 (61.61, 202.60) | | 126.03 (73.67, 236.56) | | 108.67 (52.75, 235.78) | | 146.27 (72.66, 292.41) | | <0.001 |
| Dead | 89.00 (50.64, 162.77) | | 96.47 (58.65, 168.25) | | 95.67 (55.33, 166.44) | | 82.12 (44.83, 155.84) | | 85.65 (49.82, 158.21) | | <0.001 |

Data are shown as median (IQR).

Average glucose Q1: AG <121.65mg/dL, Q2: 121.65mg/dL≤ AG <141.59mg/dL, Q3: 141.59mg/dL≤ AG <171.75mg/dL, Q4: AG ≥171.75mg/dL.

Glycemic variability Q1: GV <18.86%, Q2: 18.86%≤ GV <26.21%, Q3: 26.21%≤ GV <35.8%, Q4: GV ≥35.8%.

Group 1: AG <139.7mg/dL and GV <25.8%; Group 2: AG <139.7mg/dL and GV ≥25.8%; Group 3: AG ≥139.7mg/dL and GV <25.8%; Group 4: AG ≥139.7mg/dL and GV ≥25.8%.

P-value indicates the Kruskal-Wallis test.

ICU: Intensive Care Unit, AG: average glucose, GV: glycemic variability.

Table S9. Generalize linear models analyses for different groups and length of ICU stay in cardiac arrest patients.

|  | Model 1 | Model 2 | Model 3 | Model 4 | Model 5 | Model 6 |
| --- | --- | --- | --- | --- | --- | --- |
|  | P-value | P-value | P-value | P-value | P-value | P-value |
| **Overall** | | | | | | |
| **Average glucose** | | | | | | |
| Continuous | 0.307 | 0.257 | 0.23 | 0.053 | 0.052 | **0.017** |
| Categorical |  |  |  |  |  |  |
| Q1 | - | - | - | - | - | - |
| Q2 | **<0.001** | **<0.001** | **<0.001** | **<0.001** | **<0.001** | **<0.001** |
| Q3 | **<0.001** | **<0.001** | **<0.001** | **<0.001** | **<0.001** | **0.013** |
| Q4 | 0.174 | 0.277 | 0.501 | 0.094 | **0.007** | **<0.001** |
| **Glycemic variability** | | | | | | |
| Continuous | 0.577 | 0.606 | 0.604 | 0.701 | 0.901 | 0.511 |
| Categorical |  |  |  |  |  |  |
| Q1 | - | - | - | - | - | - |
| Q2 | **<0.001** | **<0.001** | **<0.001** | **<0.001** | **<0.001** | **<0.001** |
| Q3 | **<0.001** | **<0.001** | **<0.001** | **<0.001** | **<0.001** | **<0.001** |
| Q4 | **0.019** | **0.033** | **0.03** | 0.645 | 0.276 | 0.669 |
| **Average glucose and Glycemic variability** | | | | | | |
| Group 1 | - | - | - | - | - | - |
| Group 2 | 0.414 | 0.416 | 0.439 | 0.976 | 0.625 | 0.758 |
| Group 3 | **0.043** | **0.046** | **0.023** | **0.004** | **<0.001** | **<0.001** |
| Group 4 | 0.196 | 0.277 | 0.393 | 0.266 | 0.307 | **0.014** |
| **Survival** | | | | | | |
| **Average glucose** | | | | | | |
| Continuous | 0.929 | 0.796 | 0.738 | 0.243 | 0.223 | 0.123 |
| Categorical |  |  |  |  |  |  |
| Q1 | - | - | - | - | - | - |
| Q2 | **<0.001** | **<0.001** | **<0.001** | **<0.001** | **<0.001** | **<0.001** |
| Q3 | **<0.001** | **<0.001** | **<0.001** | **<0.001** | **<0.001** | **<0.001** |
| Q4 | **<0.001** | **<0.001** | **<0.001** | 0.099 | 0.939 | 0.497 |
| **Glycemic variability** | | | | | | |
| Continuous | **<0.001** | **<0.001** | **<0.001** | **0.005** | **0.005** | 0.792 |
| Categorical |  |  |  |  |  |  |
| Q1 | - | - | - | - | - | - |
| Q2 | **<0.001** | **<0.001** | **<0.001** | **<0.001** | **<0.001** | **<0.001** |
| Q3 | **<0.001** | **<0.001** | **<0.001** | **<0.001** | **<0.001** | **<0.001** |
| Q4 | **<0.001** | **<0.001** | **<0.001** | **0.005** | **0.006** | 0.317 |
| **Average glucose and Glycemic variability** | | | | | | |
| Group 1 | - | - | - | - | - | - |
| Group 2 | **0.004** | **0.004** | **0.013** | 0.054 | 0.052 | 0.371 |
| Group 3 | 0.412 | 0.345 | 0.501 | 0.785 | 0.643 | 0.33 |
| Group 4 | **<0.001** | **<0.001** | **<0.001** | **0.008** | **0.036** | 0.294 |
| **Dead** | | | | | | |
| **Average glucose** | | | | | | |
| Continuous | 0.126 | 0.136 | 0.128 | 0.072 | 0.088 | **0.04** |
| Categorical |  |  |  |  |  |  |
| Q1 | - | - | - | - | - | - |
| Q2 | **<0.001** | **<0.001** | **<0.001** | **<0.001** | **<0.001** | **<0.001** |
| Q3 | **0.013** | **0.011** | **0.009** | **0.02** | **0.008** | 0.081 |
| Q4 | 0.505 | 0.338 | 0.37 | **0.041** | 0.063 | **0.006** |
| **Glycemic variability** | | | | | | |
| Continuous | 0.398 | 0.441 | 0.48 | 0.267 | 0.713 | 0.457 |
| Categorical |  |  |  |  |  |  |
| Q1 | - | - | - | - | - | - |
| Q2 | **<0.001** | **<0.001** | **<0.001** | **<0.001** | **<0.001** | **<0.001** |
| Q3 | **<0.001** | **<0.001** | **<0.001** | **<0.001** | **<0.001** | **<0.001** |
| Q4 | 0.353 | 0.308 | 0.516 | 0.184 | 0.844 | 0.513 |
| **Average glucose and Glycemic variability** | | | | | | |
| Group 1 | - | - | - | - | - | - |
| Group 2 | 0.072 | 0.064 | 0.094 | 0.031 | 0.322 | 0.244 |
| Group 3 | **0.001** | **<0.001** | **<0.001** | **<0.001** | **0.001** | **<0.001** |
| Group 4 | **0.012** | **0.006** | **0.01** | **<0.001** | **0.01** | **<0.001** |

Average glucose Q1: AG <121.65mg/dL, Q2: 121.65mg/dL≤ AG <141.59mg/dL, Q3: 141.59mg/dL≤ AG <171.75mg/dL, Q4: AG ≥171.75mg/dL.

Glycemic variability Q1: GV <18.86%, Q2: 18.86%≤ GV <26.21%, Q3: 26.21%≤ GV <35.8%, Q4: GV ≥35.8%.

Group 1: AG <139.7mg/dL and GV <25.8%; Group 2: AG <139.7mg/dL and GV ≥25.8%; Group 3: AG ≥139.7mg/dL and GV <25.8%; Group 4: AG ≥139.7mg/dL and GV ≥25.8%.

Model 1: unadjusted.

Model 2: Model 1 + Gender, Age, Race, and BMI.

Model 3: Model 2 + MAP, Temperature, Heart rate, and Respiratory rate.

Model 4: Model 3 + Hypertension, Diabetes, Cerebral infarction, Acute myocardial infarction, Acute kidney injury, Liver cirrhosis, Chronic kidney disease, and Malignant tumor.

Model 5: Model 4 + WBC counts, Hemoglobin, Platelet counts, BUN, Creatinine, Total calcium, Sodium, Potassium, Chloride, and Anion gap.

Model 6: Model 5 + Mechanical ventilation, CRRT, Vasoactive-inotropic drugs, and Insulin.

ICU: Intensive Care Unit, AG: average glucose, GV: glycemic variability.


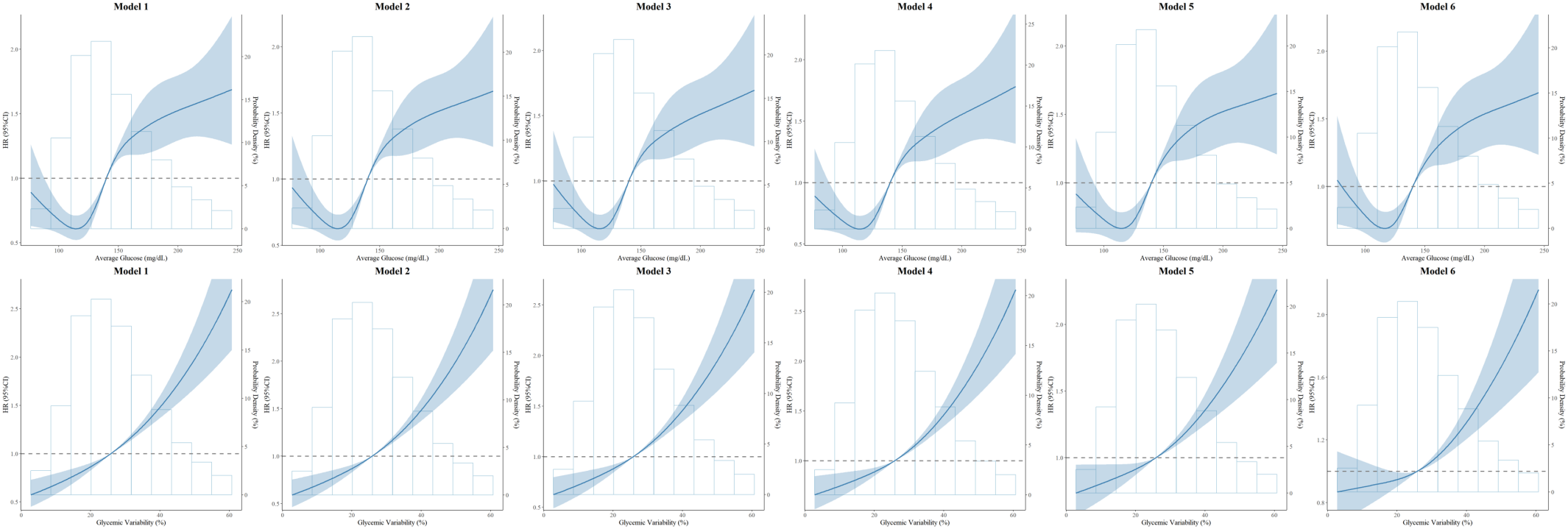


Figure S1. RCS demonstrated the relationships between AG, GV and 28-day mortality.

Model 1: unadjusted.

Model 2: Model 1 + Gender, Age, Race, and BMI.

Model 3: Model 2 + MAP, Temperature, Heart rate, and Respiratory rate.

Model 4: Model 3 + Hypertension, Diabetes, Cerebral infarction, Acute myocardial infarction, Acute kidney injury, Liver cirrhosis, Chronic kidney disease, and Malignant tumor.

Model 5: Model 4 + WBC counts, Hemoglobin, Platelet counts, BUN, Creatinine, Total calcium, Sodium, Potassium, Chloride, and Anion gap.

Model 6: Model 5 + Mechanical ventilation, CRRT, Vasoactive-inotropic drugs, and Insulin.


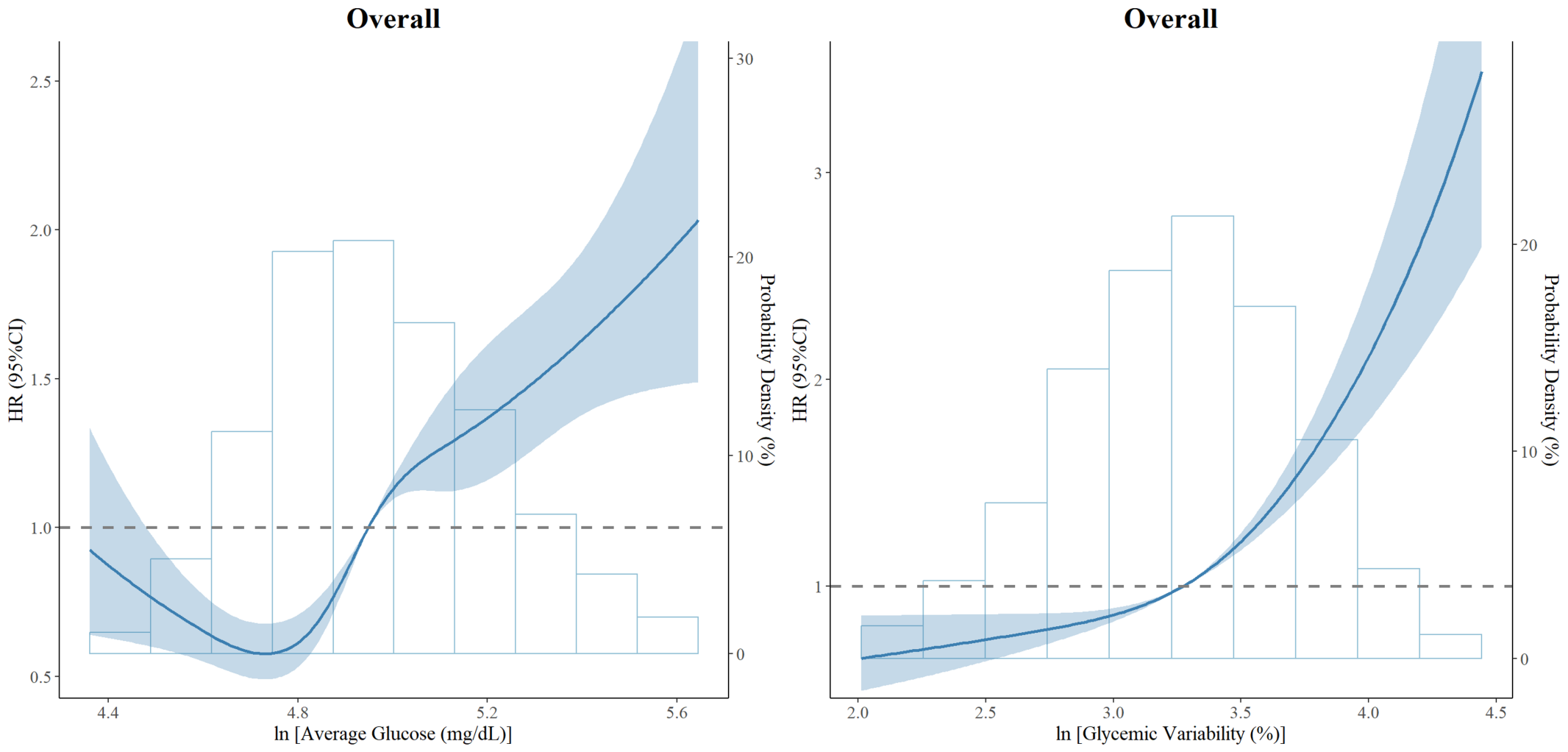


Figure S2. RCS demonstrated the relationships between ln(AG), ln(GV) and 28-day mortality.


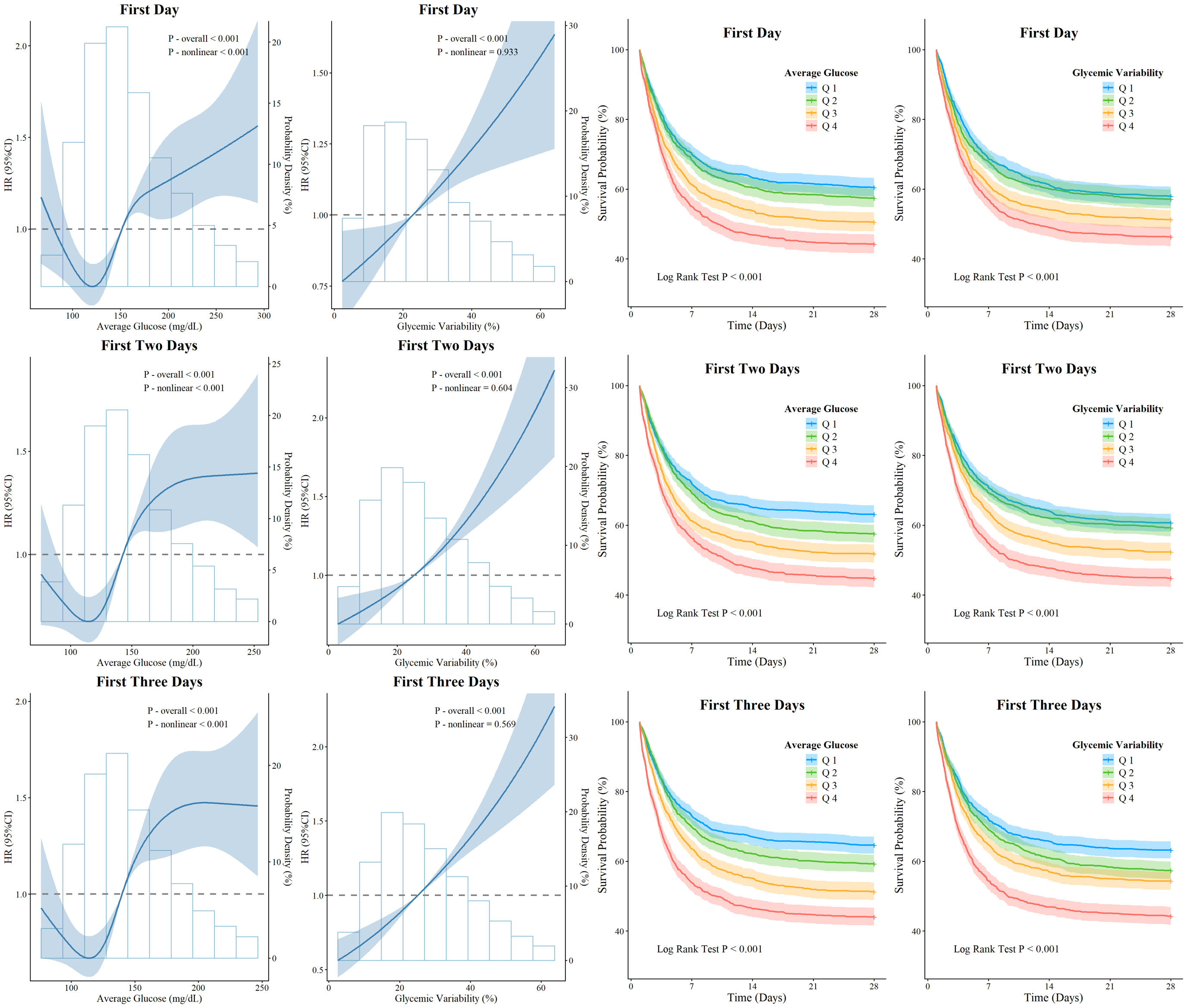


Figure S3. RCS and Kaplan-Meier curves show the associations between AG/GV during different ICU hospitalization periods and 28-day mortality.


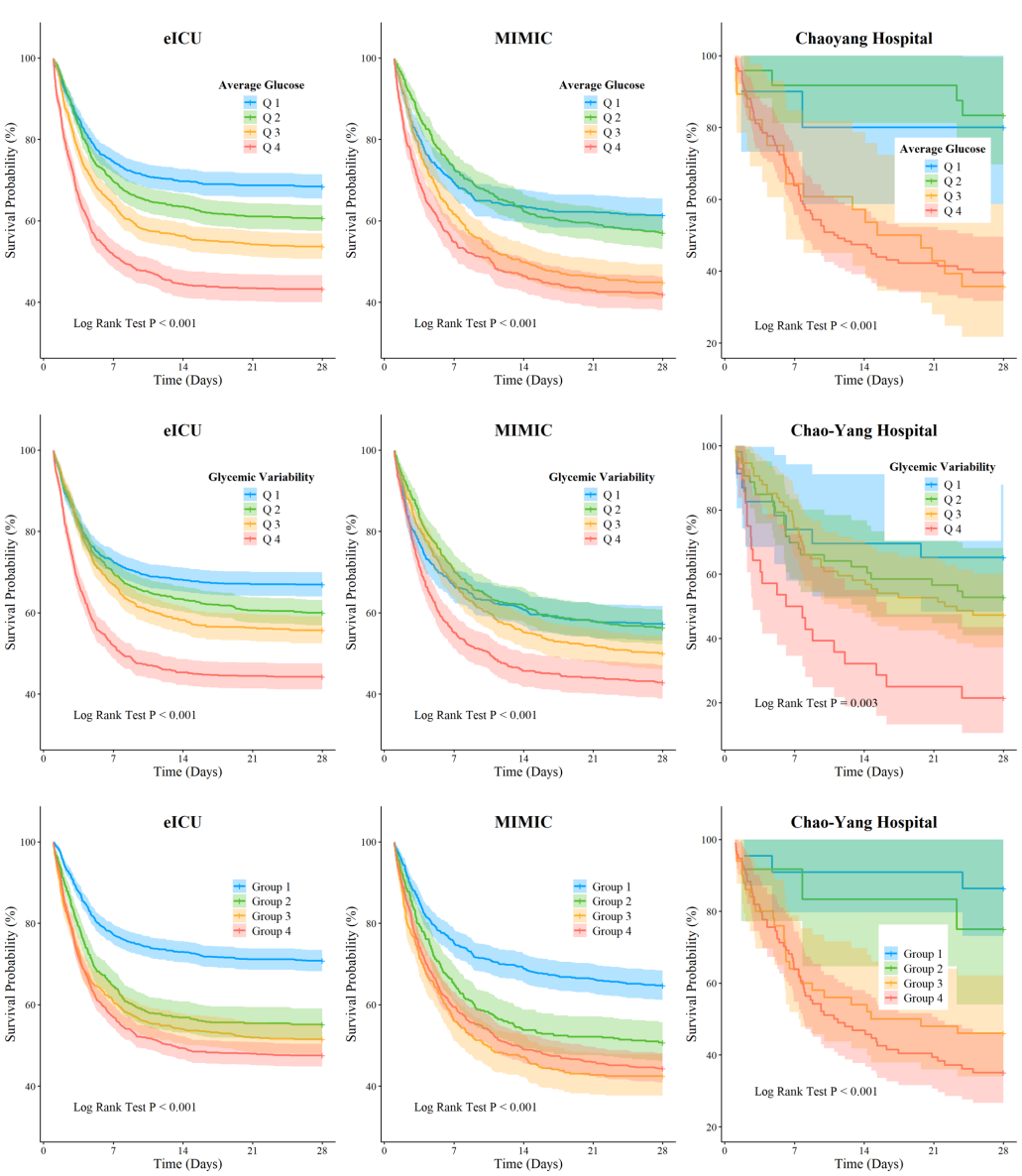


Figure S4. Kaplan-Meier curves show the associations between AG/GV and 28-day mortality in different datasets.


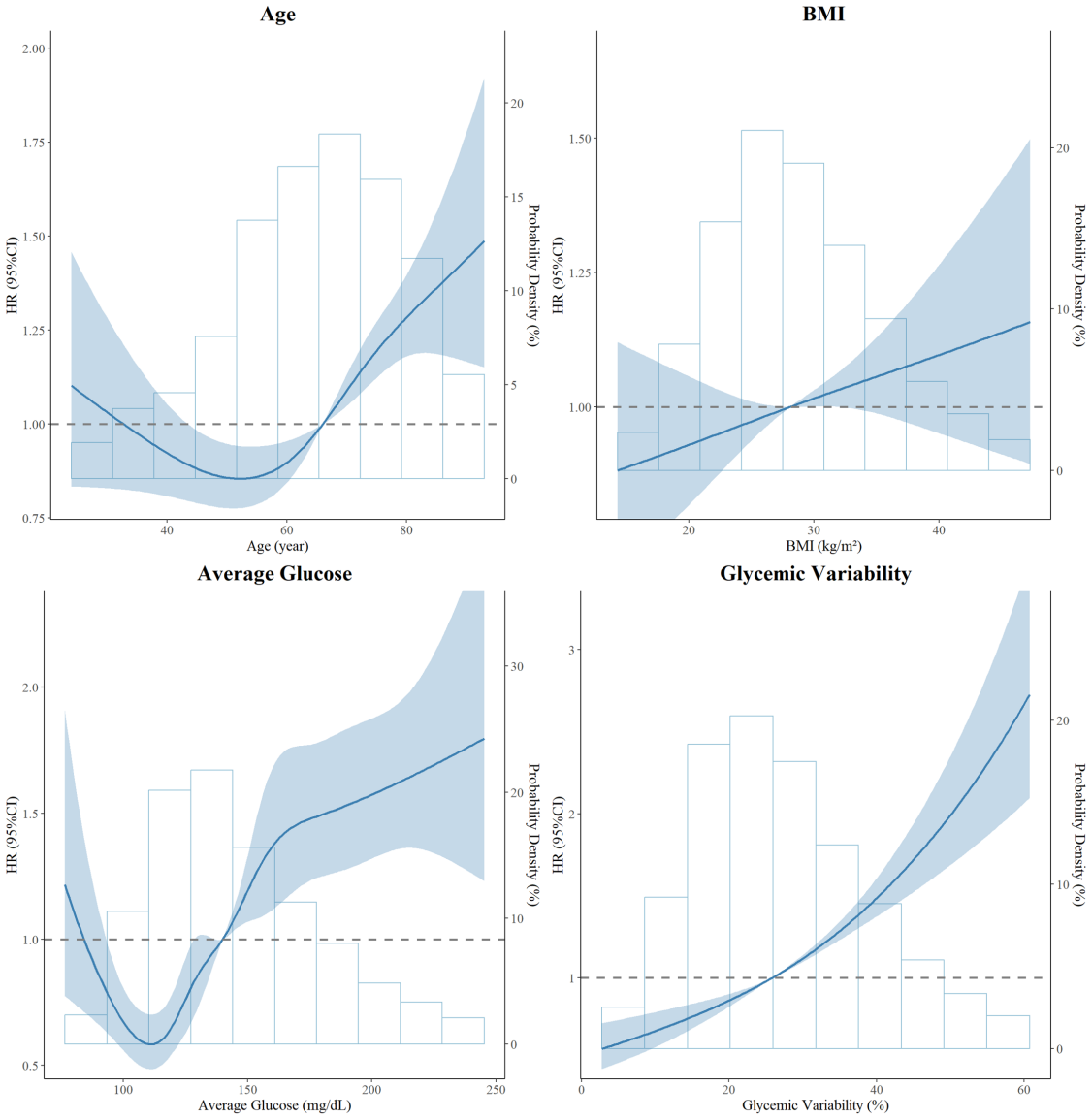


Figure S5. RCS demonstrated the relationship between continuous variables and 28-day mortality, and determined optimal thresholds for subgroup analysis.


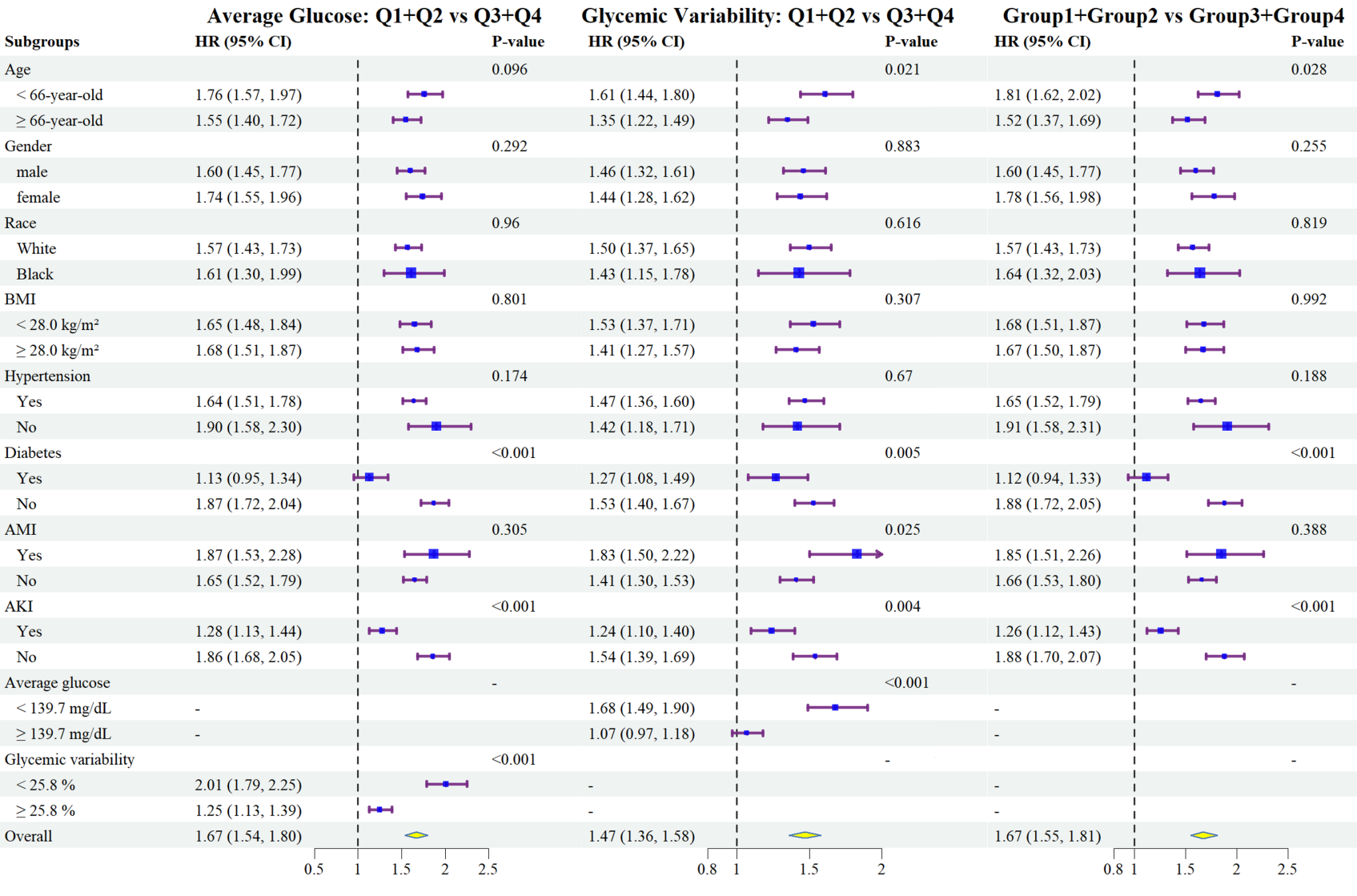


Figure S6. Subgroup analysis of the correlation between AG, GV grouping and 28-day mortality in CA patients (AG: Q1+Q2 vs. Q3+Q4; GV: Q1+Q2 vs. Q3+Q4; AG+GV: Group1+Group2 vs. Group3+Group4).


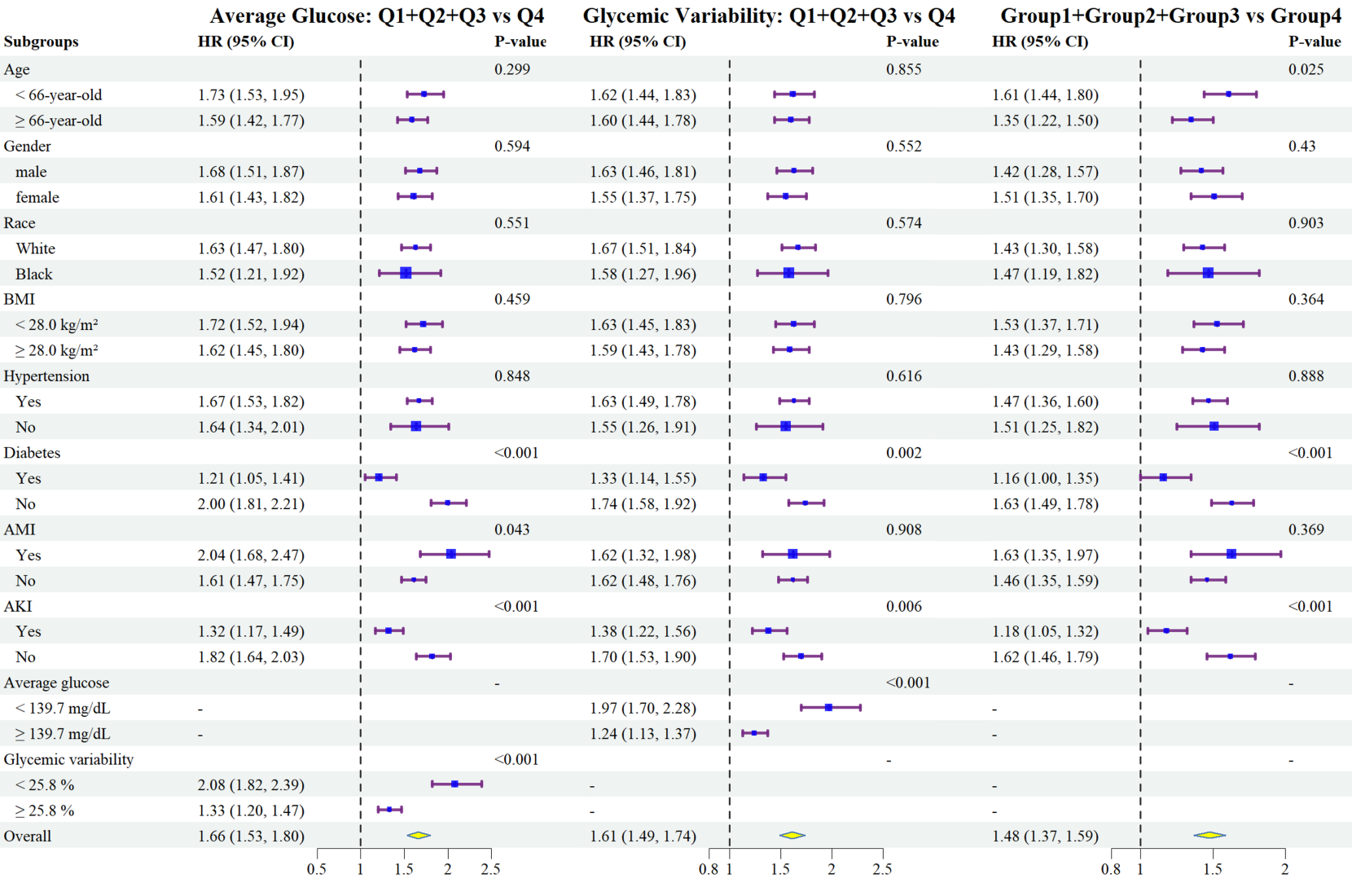


Figure S7. Subgroup analysis of the correlation between AG, GV grouping and 28-day mortality in CA patients (AG: Q1+Q2+Q3 vs. Q4; GV: Q1+Q2+Q3 vs. Q4; AG+GV: Group1+Group2+Group3 vs. Group4).


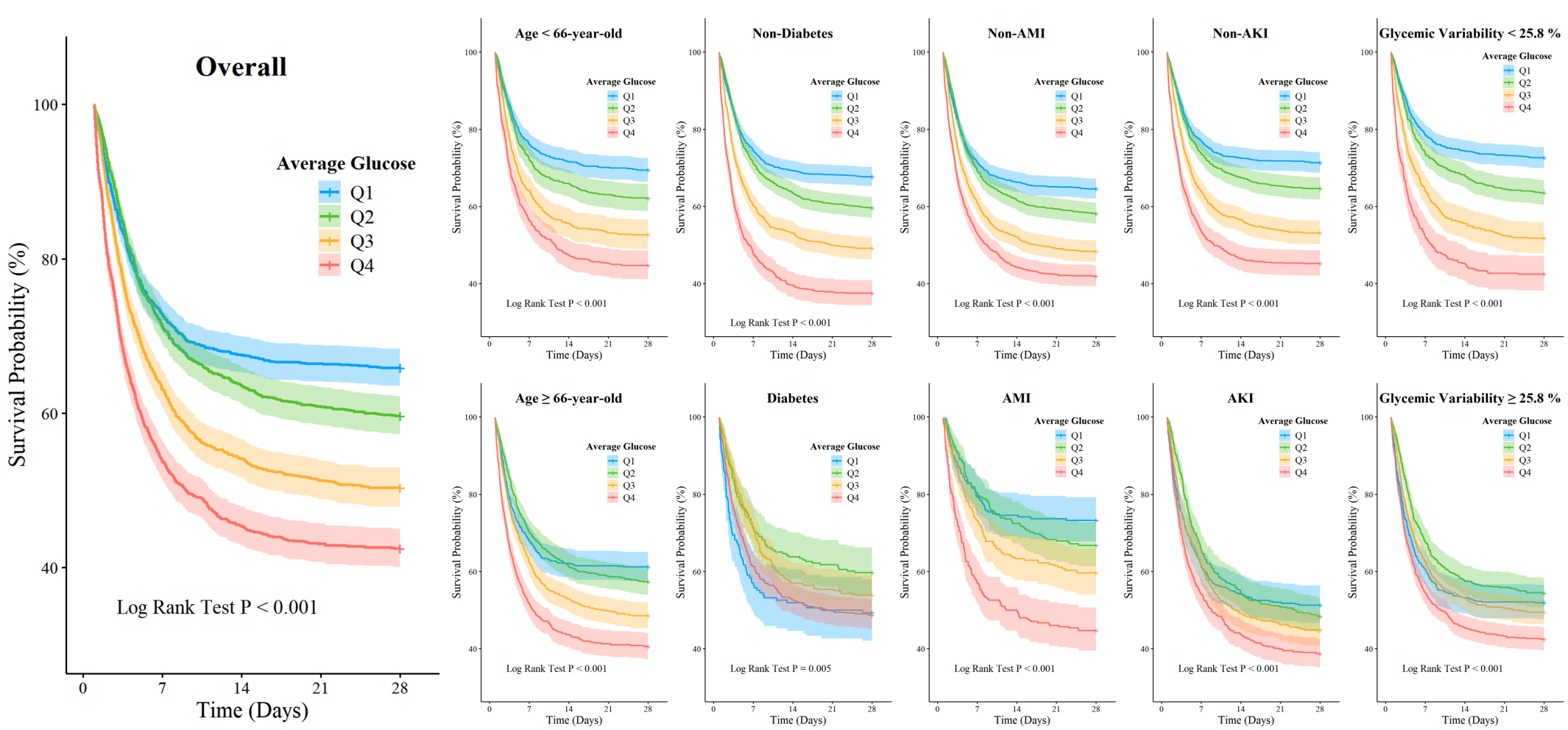


Figure S8. Kaplan-Meier curves of 28-day mortality were plotted based on the quartiles of AG in different subgroups.

**
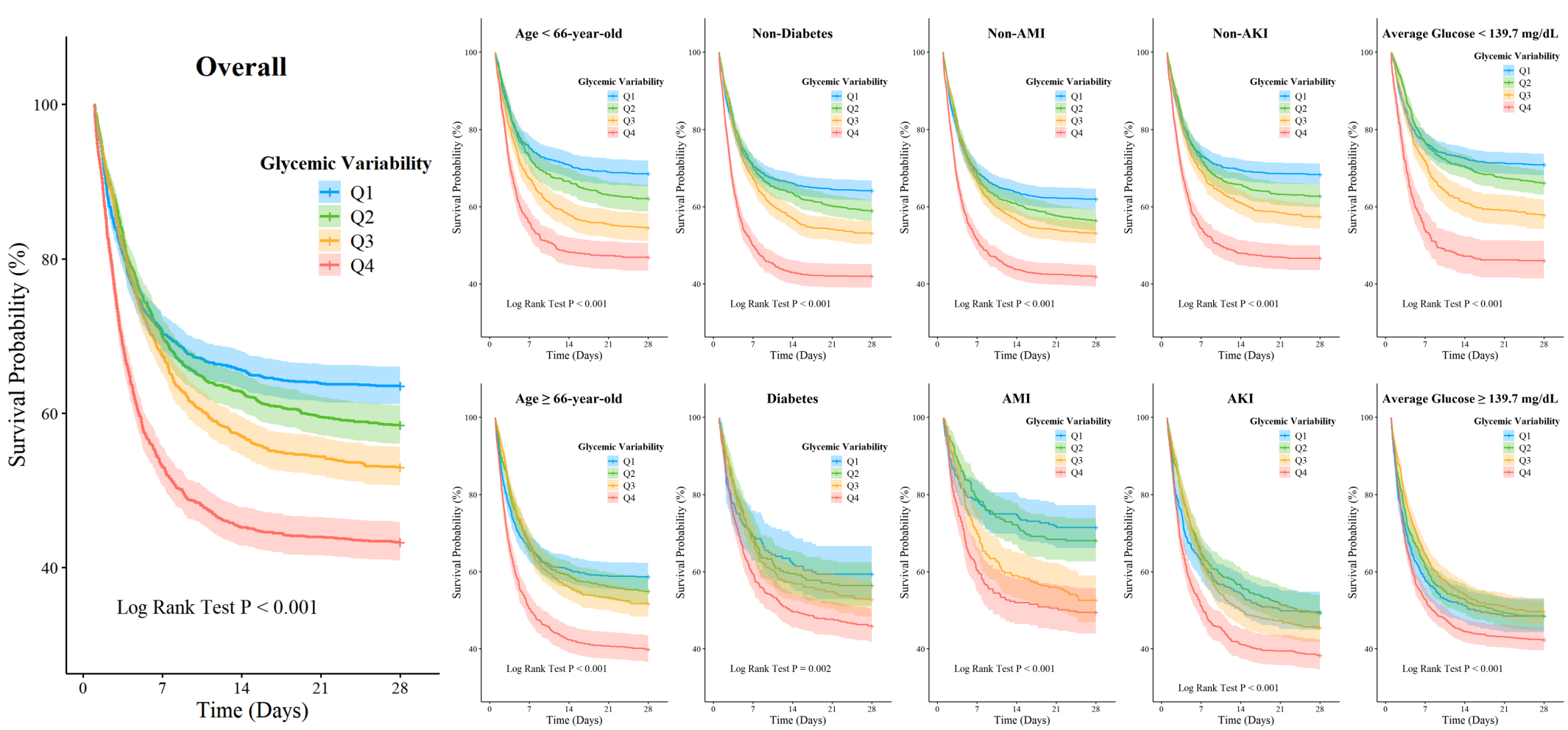
**

Figure S9. Kaplan-Meier curves of 28-day mortality were plotted based on the quartiles of GV in different subgroups.

**
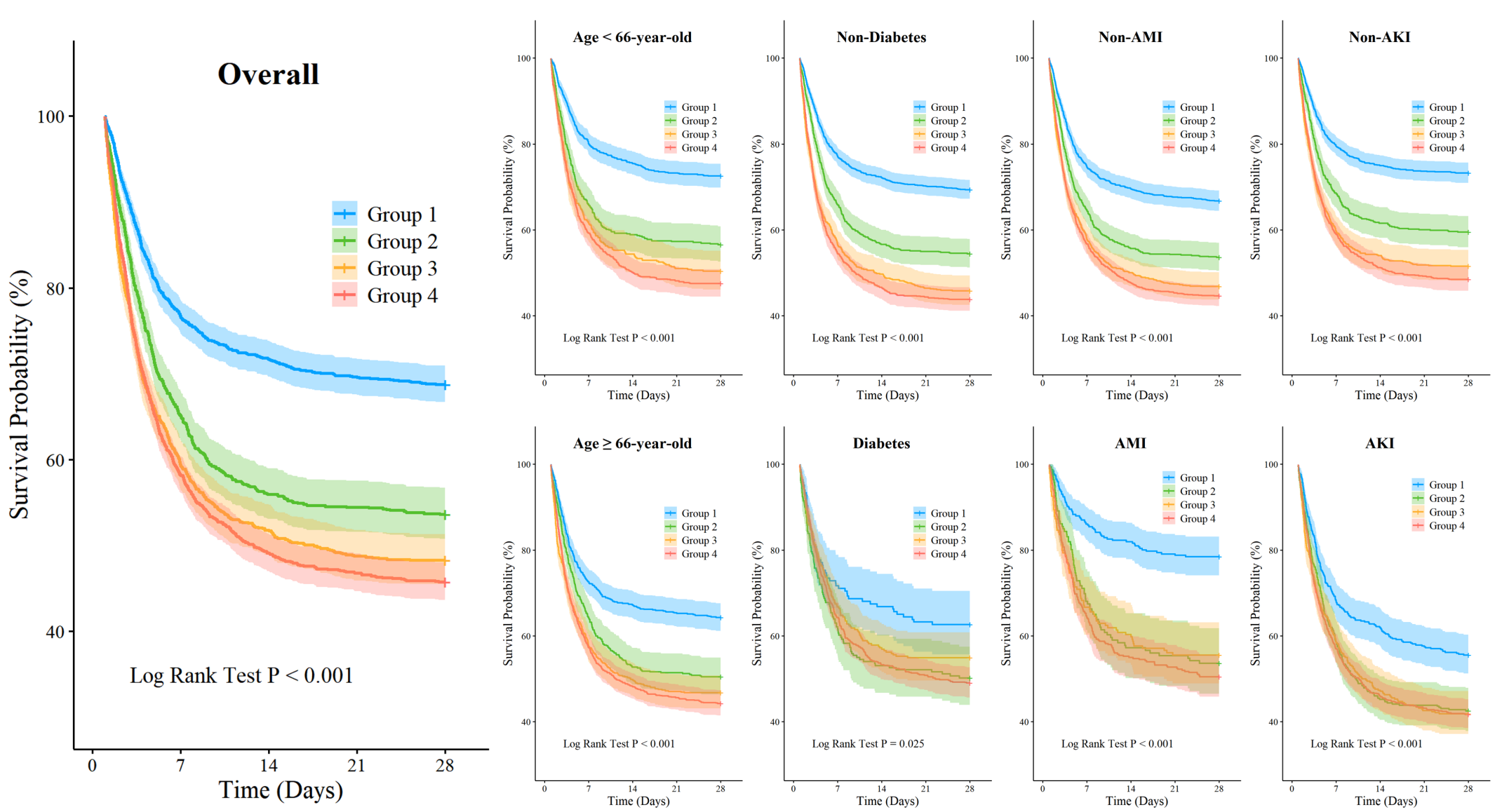
**

Figure S10. Kaplan-Meier curves of 28-day mortality were plotted according to AG and GV groups in different subgroups.

**
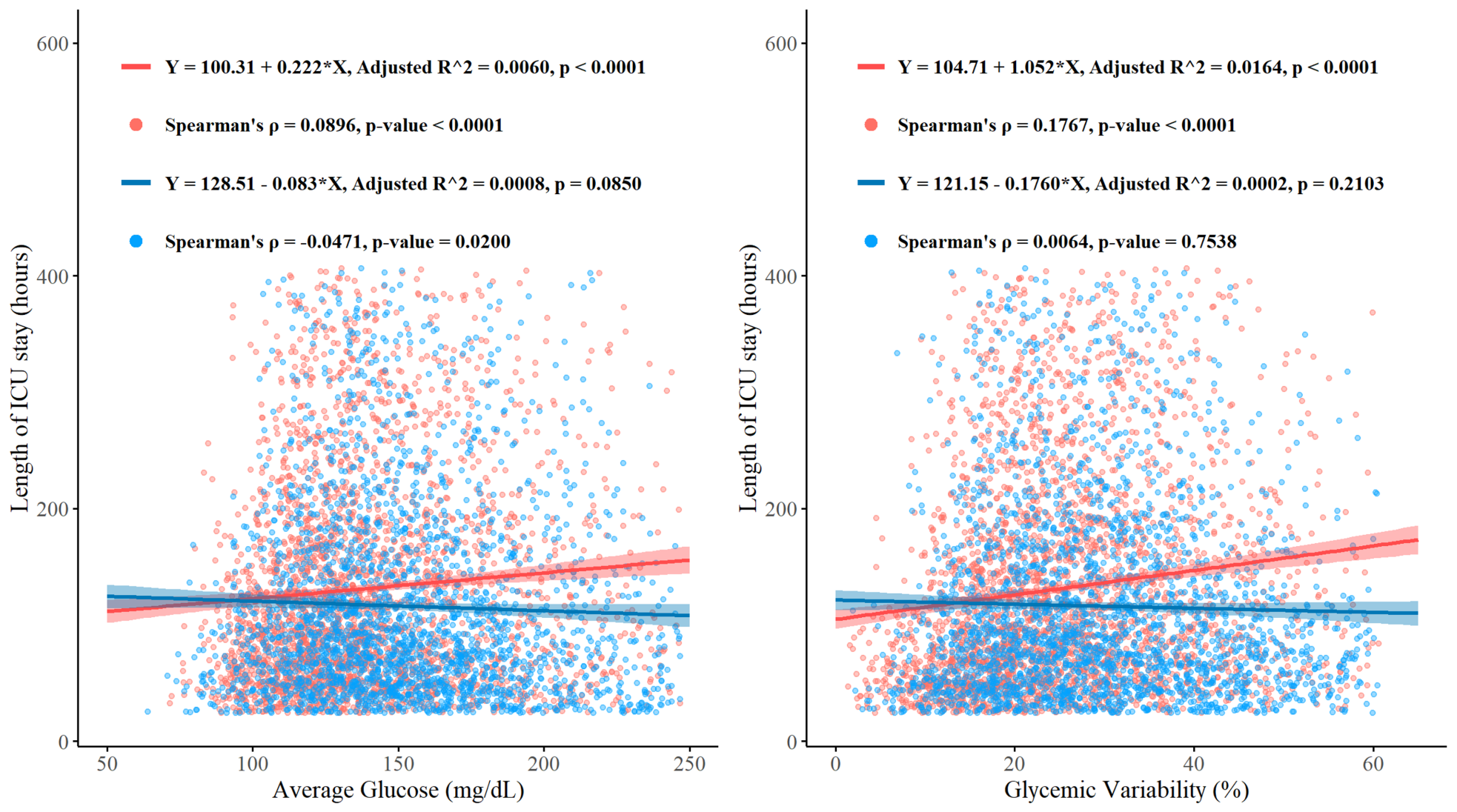
**

Figure S11. The correlation between AG and GV with the LOS in the ICU.
